# Spatiotemporal Dynamic Immunomodulation by Infection‐Mimicking Gels Enhances Broad and Durable Protective Immunity Against Heterologous Viruses

**DOI:** 10.1002/advs.202412116

**Published:** 2025-01-13

**Authors:** Seung Mo Jin, Ju Hee Cho, Yebin Seong, Wijesinghe Arachchilage Gayan Chathuranga, Yejin Gwak, Young‐Woock Noh, Min‐Ho Lee, Sang‐Seok Oh, Jin‐Ho Choi, Jong‐Soo Lee, Yong Taik Lim

**Affiliations:** ^1^ SKKU Advanced Institute of Nanotechnology (SAINT) Department of Nano Engineering Department of Nano Science and Technology School of Chemical Engineering Biomedical Institute for Convergence at SKKU Sungkyunkwan University 2066 Seobu‐ro, Jangan‐gu Suwon Gyeonggi‐do 16419 Republic of Korea; ^2^ College of Veterinary Medicine Chungnam National University Daejeon 34134 Republic of Korea; ^3^ New Drug Development Center Osong Medical Innovation Foundation Cheongju 28160 Republic of Korea

**Keywords:** adjuvant, drug delivery, immunomodulation, influenza, nanovaccine, SARS‐CoV‐2

## Abstract

Despite their safety and widespread use, conventional protein antigen‐based subunit vaccines face significant challenges such as low immunogenicity, insufficient long‐term immunity, poor CD8^+^ T‐cell activation, and poor adaptation to viral variants. To address these issues, an infection‐mimicking gel (IM‐Gel) is developed that is designed to emulate the spatiotemporal dynamics of immune stimulation in acute viral infections through in situ supramolecular self‐assembly of nanoparticulate‐TLR7/8a (NP‐TLR7/8a) and an antigen with tannic acid (TA). Through collagen‐binding properties of TA, the IM‐Gel enables sustained delivery and enhanced retention of NP‐TLR7/8a and protein antigen in the lymph node subcapsular sinus of mice for over 7 days, prolonging the exposure of vaccine components in both B cell and T cell zones, leading to robust humoral and cellular responses. The IM‐Gel system with the influenza A antigen confers cross‐protection against multiple influenza subtypes (H1N1, H5N2, H3N2, H7N3, and H9N2) with long‐term immune responses. Combination of the IM‐Gel with the SARS‐CoV‐2 spike protein also elicits strong cross‐reactive antibody responses against multiple SARS‐CoV‐2 variants (Alpha, Beta, NY510+D614G, Gamma, Kappa, and Delta). The IM‐Gel, as a programmable immunomodulatory material, provides a vaccine design principle for the development of next‐generation universal vaccines that can elicit broad and durable protective immunity against emerging viruses.

## Graphical abstract

An infection‐mimicking gel (IM‐Gel) is developed that emulates the spatiotemporal immune dynamics of acute viral infections. By enabling sustained lymph node retention of vaccine components, IM‐Gel enhances humoral and cellular immunity, offering cross‐protection against influenza subtypes and SARS‐CoV‐2 variants. This programmable platform highlights a next‐generation approach for developing universal vaccines with broad and durable immunity.

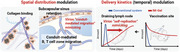

## 1 Introduction

Subunit vaccines, often combined with adjuvants to improve immunogenicity, are among the safest and most widely used types of vaccines and are highly effective in eliciting both humoral and cellular immune responses.^[^ ^1^ ^]^ Therefore, the development of safe and effective subunit vaccines against various diseases, such as influenza^[^ ^2^ ^]^ and SARS‐CoV‐2,^[^ ^3^ ^]^ is expected to be a significant advancement in modern public health. Despite the successful development of subunit vaccines and their clinical use, they face challenges in adapting to variants, generating long‐term immunity and T cell responses, and may not induce the range of immune responses that are stimulated by natural infection.^[^ ^4^ ^]^ Close observation of the natural infection patterns of viruses reveals unique macroscopic characteristics that are not replicable with conventional subunit vaccines. During acute infection, viral exposure occurs downstream of the draining lymph nodes, which is a key site from the beginning of the immune response to develop humoral and cellular immunity.^[^ ^5^ ^]^ To evoke mature effector B and T cells, the virus first demonstrates time‐dependent self‐replication, peaking at days 5–7 and continuing over 14 days.^[^ ^6^ ^]^ During this period, the virus continuously exposes its antigens and danger signals to the innate and adaptive immune systems. This contrasts sharply with conventional subunit vaccines, which are rapidly cleared within 1–2 days from draining lymph nodes (dLN). Furthermore, during acute infection, the virus can flow to the paracortex region in the dLN by penetrating LN conduits and infecting paracortical LN resident dendritic cells (DCs) within the T cell zone, leading to rapid and direct CD8^+^ T cell priming.^[^ ^7^ ^]^ However, most conventional vaccines struggle to reach the paracortex because of limited access across the subcapsular sinus (SCS),^[^ ^8^ ^]^ resulting in poor elicitation of CD8^+^ T cell immunity.^[^ ^9^ ^]^ Therefore, there is an urgent need to develop a novel, next‐generation biomaterial‐based vaccine platform that mimics acute viral infections.

**Figure 1:**
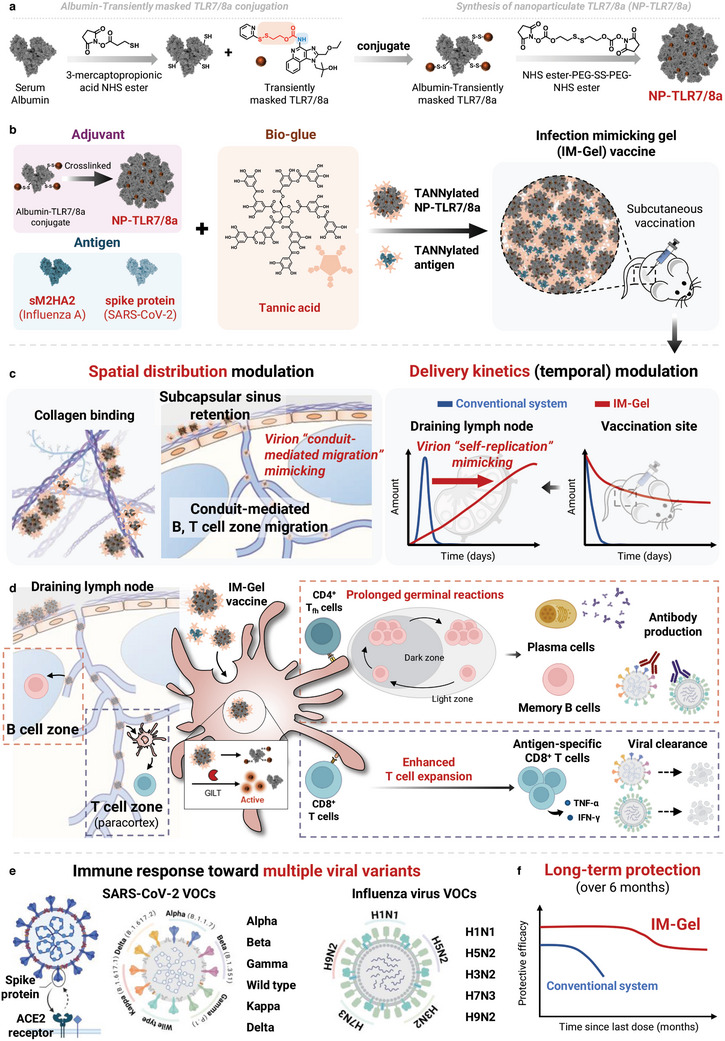
Infection mimicking gel (IM‐Gel) vaccine spatiotemporally modulating immune responses in draining lymph node (dLN) for enhanced broad and durable protective immunity. a) Synthesis of nanoparticulate TLR7/8a (NP‐TLR7/8a). b) Components and synthesis of IM‐Gel vaccine. c) Delivery kinetics (temporal) and spatial distribution of the IM‐Gel vaccine in the dLN. d) Mechanism of humoral and cellular immune response generation in dLN. e) IM‐Gel for enhanced broad and durable protective immunity against SARS‐CoV‐2 and Influenza virus.

Since collagen type I is distributed from the LN SCS along the inside of the conduit,^[^ ^10^, ^11^ ^]^ we believe that utilizing collagen binding for retention and sustained delivery to the T and B cell zones could be an effective strategy. Tannic acid (TA), which is classified as generally recognized as safe (GRAS) by the Food and Drug Administration (FDA), is rich in phenolic (hydroxyl‐rich) groups, enabling the formation of hydrogen bonds and hydrophobic interactions with proteins,^[^ ^12^, ^13^ ^]^ including proline‐rich proteins such as collagen.^[^ ^14^, ^15^ ^]^ Therefore, we hypothesized that the collagen‐binding effect of TA, along with its ability to form supramolecular coacervates with proteins, can be used to mimic the spatiotemporal aspects of acute infection. We have precisely designed an engineering‐based “infection‐mimicking” biomimetic immune‐material, termed infection‐mimicking‐gel (IM‐Gel). To enable the efficient delivery of the small molecular TLR7/8a to the LNs while preventing systemic leakage, we employed two strategies: protein‐small molecule conjugation and nanoparticle‐based delivery (**Figure** **1**a). IM‐Gel was formulated through the in situ supramolecular self‐assembly of nanoparticulate‐TLR7/8a (NP‐TLR7/8a) with TA, targeting collagen in the LN SCS, enabling the sustained delivery of a low dose of TANNylated NP‐TLR7/8a and its dissociation through the conduit to the paracortex for over 7 days, exposing their antigen and danger signals for prolonged periods, and elucidating antigen‐specific antibody and CD8^+^ T cell responses (Figure 1b–d).

Influenza viruses^[^ ^16^ ^]^ and SARS‐CoV‐2^[^ ^17^ ^]^ continuously undergo antigenic drift, resulting in new variants that limit the scope and effectiveness of current vaccines. Therefore, there is a critical need to develop cross‐reactive vaccines that provide broad protection against multiple variant virus strains. The influenza matrix protein 2 (M2) and hemagglutinin fusion peptide (HA2) are well‐conserved regions across influenza strains, making them attractive antigen candidates for universal vaccines.^[^ ^18^ ^]^ However, despite testing several hundred adjuvants over the past decades, only a few have been approved for human use owing to their intrinsically low immunogenicity and limited efficacy,^[^ ^19^ ^]^ and until recently, aluminum‐based mineral salts or MF59, which rarely demonstrate acute infection‐like properties, have been most widely used for human vaccines.^[^ ^19^ ^]^ Therefore, we incorporated M2HA2 of influenza and the spike protein of SARS‐CoV‐2 into the IM‐Gel system and explored its potential as a universal vaccine against influenza or SARS‐CoV‐2, broadening reactivity and durability across variants, and promoting both antibody and CD8^+^ T cell responses (Figure 1e).

## 2 Results and Discussion

### 2.1 Synthesis and Physiological Characterization of NP‐TLR7/8a and IM‐Gel (NP‐TLR7/8a)

**Figure 2:**
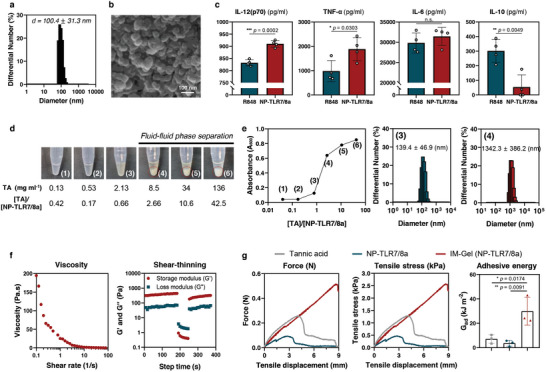
Physiological characterization of NP‐TLR7/8a and IM‐Gel (NP‐TLR7/8a). a) Dynamic light scattering analysis of NP‐TLR7/8a particle size distribution. b) Representative scanning electron micrograph of the nanoparticle structure of NP‐TLR7/8a. c) Concentrations of secreted proinflammatory cytokines (IL‐12(p70), TNF‐α, and IL‐6) and an anti‐inflammatory cytokine (IL‐10) after treatment with R848 or NP‐TLR7/8a for 36 h in BMDCs (*n* = 4). d,e) Formation of coacervates (fluid–fluid phase separated) depending on the stoichiometric ratio of tannic acid (TA) and NP‐TLR7/8a. Formation of coacervates verified by visualization (d) and by turbidity measurement at A600 and particle size distribution (e). Red dotted line circle indicates coacervate. f) Rheological properties of IM‐Gel (NP‐TLR7/8a). Viscosity versus shear rate (left) and shear thinning property (right). G′ and G″ were measured with alternating shear strains of 0.2 and 500% strain at a frequency of 1 Hz (lower). g) Bioadhesive strength measurements by lap‐shear test (adhesive energy, G_ad_) (*n* = 3). All data are presented as the mean ± s.d. Statistical significance was evaluated by one‐way ANOVA with Tukey's multiple comparison test in (g) and by an unpaired two‐tailed *t* test in (c). *p* values: NS, not significant; *p < 0.05, **p < 0.01, ***p < 0.001, ****p < 0.0001.

We conjugated transiently masked TLR7/8a (Figures S1–S4, Supporting Information) with serum albumin and induced their nanoparticulation via a covalent crosslinker. The size distribution of NP‐TLR7/8a was confirmed to be between 90 and 110 nm, using DLS, scanning electron microscopy (SEM) and atomic force microscopy (AFM) (**Figure** **2**a,b; Figure S5, Supporting Information). Previous studies have demonstrated that upon endocytosis in antigen‐presenting cells, the gamma interferon‐inducible lysosomal thiol reductase (GILT)‐responsive linker of transiently masked TLR7/8a undergoes intracellular exposure and cleavage, leading to time‐dependent activation (Figure 1d).^[^ ^20^ ^]^ This durable TLR stimulation via transiently masked TLR7/8a, which could suppress TLR tolerance, induced the production of higher level of proinflammatory cytokines (IL‐6, TNF‐α, and IL‐12(p70)) compared to R848 treatment, while secreting lower levels of the anti‐inflammatory cytokine IL‐10 (Figure 2c; Figure S6, Supporting Information).

To regulate the spatiotemporal dynamics of NP‐TLR7/8a, we mixed them stoichiometrically with TA, inducing physical crosslinking and forming an IM‐Gel (NP‐TLR7/8a) (Figure 1b). As the concentration of TA increased, it covered the surface of NP‐TLR7/8a. Beyond a critical value of the [TA]/[t‐CNV] ratio, fluid–fluid phase separation occurred, leading to the formation of a visible coacervate (Figure 2d). This phenomenon was confirmed by a significant increase in the absorbance (A_600_; turbidity assay) and particle size (Figure 2e). The structure of IM‐Gel (NP‐TLR7/8a) was confirmed by SEM images (Figure S7, Supporting Information). Coacervate‐like IM‐Gel (NP‐TLR7/8a) exhibited high viscosity (≈200 Pa⋅s) and demonstrated shear‐thinning behavior, allowing syringe injection as a viscous liquid (with G″ > G′) under stress (Figure 2f). Upon stress removal, it formed a depot at the site of injection by transitioning back to a gel‐like state (with G′ > G″) (Figure 1k). Furthermore, owing to surface modification with TA, IM‐Gel (NP‐TLR7/8a) displayed robust bioadhesiveness and notably greater adhesive energy (G_ad_) than NP‐TLR7/8a and soluble TA (Figure 2g). For further in vivo applications, we evaluated the short‐ and long‐term biosafety of IM‐Gel by assessing body weight changes and serum IL‐6 cytokine levels (short‐term), ALT and AST activities (long‐term), and histological sections of various organs (lung, liver, kidney, and spleen) at 7 days post‐immunization (Figures S8 and S9, Supporting Information). No significant toxicity was observed across all four indicators, indicating good biocompatibility of safety of IM‐Gel in vivo.

### 2.2 Acute Infection‐Like Spatiotemporal Organization of Antigen and Adjuvant in dLN

**Figure 3:**
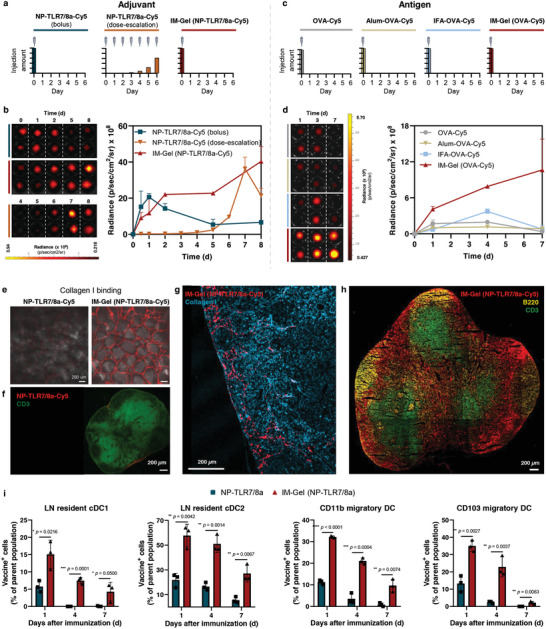
Acute infection‐like spatiotemporal organization of antigen and adjuvant in draining lymph nodes. a,b) Lymph node delivery kinetics of adjuvant systems after subcutaneous vaccination. Injection schedule and dose of each adjuvant system (a). In vivo imaging system (IVIS) image and average radiance of fluorescent signal in the draining lymph node (dLN) over time (b). c,d) Lymph node delivery kinetics of antigen with or without various adjuvant systems after subcutaneous vaccination. Injection schedule and dose of each adjuvant system (c). IVIS image and average radiance of fluorescent signal in the dLN over time (d). e) Collagen I binding ability verified through representative confocal microscopy image of SpongeCol^®^ (a type I collagen sponge with columnar pore architecture) after incubation with NP‐TLR7/8a‐Cy5 or IM‐Gel (NP‐TLR7/8a) for 1 h and washed vigorously. Scale bar, 200 µm. f) Representative confocal microscopy images of spatial localization of NP‐TLR7/8a‐Cy5 in dLN lining in subcapsular sinus (SCS) 1 day after subcutaneous vaccination. Scale bar, 200 µm. g) Representative confocal microscopy images of spatial localization of IM‐Gel (NP‐TLR7/8a‐Cy5) in dLN through the conduit (collagen I‐stained) 7 days after subcutaneous vaccination. Scale bar, 200 µm. h) Representative confocal microscopy images of intra‐LN delivery of IM‐Gel (NP‐TLR7/8a‐Cy5) into T cell zone (CD3) and B cell zone (B220) after subcutaneous vaccination. Scale bar, 200 µm. i) Percentage of vaccine^+^ dendritic cells (DCs) in the dLN at 1, 4, or 7 days after subcutaneous vaccination of NP‐TLR7/8a‐Cy5 or IM‐Gel (NP‐TLR7/8a‐Cy5). APCs are defined as LN resident cDC1s (CD11b^−^CD11c^+^CD8^+^), LN resident cDC2s (CD11b^+^CD11c^+^CD8^−^CD4^+^CD103^−^), CD11b migratory DCs (CD11b^+^CD11c^+^CD8^−^CD4^−^CD103^−^), and CD103 migratory DCs (CD11b^−^CD11c^+^CD8^−^CD4^−^CD103^+^) (*n* = 3). All data are presented as the mean ± s.d. Statistical significance was evaluated by an unpaired two‐tailed *t* test in i. *p* values: NS, not significant; *p < 0.05, **p < 0.01, ***p < 0.001, ****p < 0.0001.

To elucidate the delivery kinetics of the vaccines after vaccination, we monitored their signals at both the injection site (local) and dLN using an in vivo imaging system (IVIS). Because of its high bioadhesiveness, IM‐Gel (NP‐TLR7/8a‐Cy5) was detected at the injection site over four days, while NP‐TLR7/8a‐Cy5 alone dissipated within one day (Figure S10, Supporting Information). We also observed that bioadhesiveness of IM‐Gel (NP‐TLR7/8a) recruits immune cells to local injection sites for over 3 days (Figure S11, Supporting Information). To simulate the kinetics of virus self‐replication in acute infection, we included a dose‐escalation group that artificially mimicked the kinetics of virus self‐replication by administering injections every day for 6 days with exponentially increasing doses, while the total summed dose was kept the same as that of the bolus group.^[^ ^21^ ^]^ The NP‐TLR7/8a‐Cy5 (dose‐escalation) signal at the local injection site was present on the day of vaccination but quickly diminished by the next day. Next, we compared their delivery to draining lymph nodes (**Figure** **3**a,b). The NP‐TLR7/8a‐Cy5 (bolus) signal was detected in the dLN on day 1, but rapidly decreased as it flushed away, demonstrating the intrinsic limitations of conventional vaccine systems. However, incorporating NP‐TLR7/8a‐Cy5 into the IM‐Gel resulted in a continuously increasing signal in the dLN, which was sustained for over 6–8 days. NP‐TLR7/8a‐Cy5 (dose escalation) was detected after 4th injection, with the signal increasing exponentially over three sequential vaccinations. Interestingly, the maximum signal on day 7 was slightly higher than that of NP‐TLR7/8a‐Cy5 (bolus) on day 1. We also observed time‐dependent biodistribution of IM‐Gel in different organs after subcutaneous injection (Figure S12, Supporting Information).

Next, we incorporated a model protein antigen (OVA) into the IM‐Gel system through simple mixing and observed its delivery kinetics in the dLN (Figure 3c,d), comparing them with commercial adjuvant systems (alum and Incomplete Freund's adjuvant (IFA)) known to induce immunity by creating a depot and local retention of OVA.^[^ ^22^ ^]^ Although alum and IFA are known to deposit protein antigens at the injection site, their continuous delivery to dLNs is substantially limited. In contrast, the IM‐Gel (OVA‐Cy5) proved to be very effective in continuously delivering OVA to the dLN over 7 days. We hypothesized that the continuous delivery from the IM‐Gel was due to both sustained delivery from the local injection site (≈4 days) and the LN‐binding mechanism of the TANNylated vaccine.

We assumed that the strong binding affinity of the IM‐Gel (NP‐TLR7/8a) for type I collagen differentiates its spatiotemporal delivery (Figures 1c and 3e). On day 7, a portion of the IM‐Gel (NP‐TLR7/8a‐Cy5) was aligned with collagen I in the SCS, with a considerable amount penetrating the intra‐LNs following the collagen I conduit structure (Figure 3g). This contrasted sharply with NP‐TLR7/8a, which was mostly located outside the SCS and was flushed away on day 1 (Figure 3f). Specifically, the IM‐Gel (NP‐TLR7/8a‐Cy5) that entered the conduit led to the intra‐follicle and paracortical (T cell) areas (Figure 3h). In the intrafollicular area, the IM‐Gel (NP‐TLR7/8a‐Cy5) can be directly captured by cognate B cells and enters the B‐cell follicle to generate antibody responses.^[^ ^8^, ^23^ ^]^ In the paracortex, it is crucial for the vaccine to be taken up by LN‐resident cDC1 and cDC2 to generate antigen‐specific CD4^+^ and CD8^+^ T cells responses.^[^ ^7^, ^10^ ^]^ Indeed, IM‐Gel (NP‐TLR7/8a) showed more effective uptake by LN‐resident cDC1 and cDC2 than NP‐TLR7/8a (Figure 3i; Figures S13 and S14, Supporting Information). Additionally, IMGel (NP‐TLR7/8a) demonstrated a higher uptake by migratory DC than NP‐TLR7/8a, likely because of the depot effect formed at the local injection site (Figure 3i).

### 2.3 Dynamic Innate and Adaptive Immune Responses via Acute Infection‐Like Vaccination

**Figure 4:**
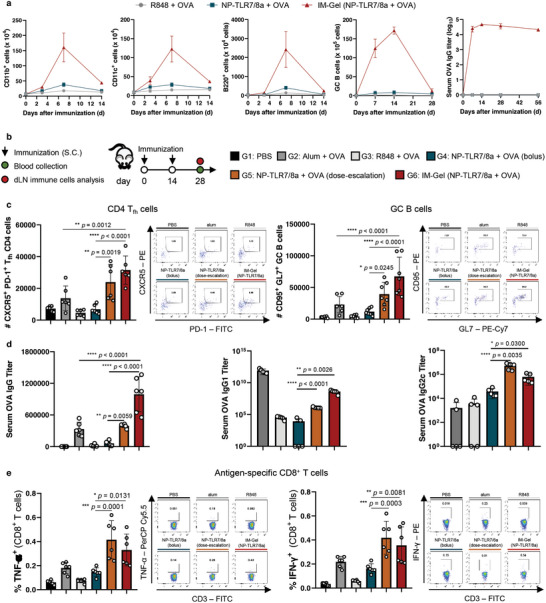
Dynamic innate and adaptive immune responses via acute infection‐like vaccination. a) Longitudinal analysis of the number of immune cells (CD11b^+^ cells, CD11c^+^ cells, B220^+^ cells, and GC B cells) and serum OVA IgG titer (*n* = 3). Mice were subcutaneously immunized with indicated samples (OVA 10 µg, TLR7/8a 30 nmol). b–e) Analysis of human and cellular immune responses after prime‐boost vaccination. Indicated samples (OVA 10 µg, TLR7/8a 30 nmol) were injected two times with a 14‐day interval. Blood and dLN were collected for immune analysis 14 days after boosting (b). Number of CD4^+^ T_fh_ cells (CXCR5^+^ PD‐1^+^ CD4^+^ cells) (*n* = 6) and GC B cells (CD95^+^ GL7^+^ B220^+^ IgD^−^ cells) (*n* = 7) in dLN (c). Serum OVA‐specific IgG, IgG1, and IgG2 titer (*n* = 6 for IgG, *n* = 5 for IgG1 and IgG2) (d). Antigen‐specific CD8^+^ T cells after SIINFEKL peptide restimulation for 6 h (*n* = 6) (e). All data are presented as the mean ± s.d. Statistical significance was evaluated by one‐way ANOVA with Tukey's multiple comparison test in c‐e. *p* values: NS, not significant; *p < 0.05, **p < 0.01, ***p < 0.001, ****p < 0.0001.

Given the evidence of the spatiotemporal lymph node delivery of the IM‐Gel (NP‐TLR7/8a), we performed longitudinal analyses after immunization to observe the priming and duration of immune responses in the dLN over time. Similar to the dosing kinetics, the population of representative innate immune cells, such as CD11b^+^ and CD11c^+^ cells, continuously increased over a week after vaccination with the IM‐Gel (NP‐TLR7/8a) (**Figure** **4**a). The number of B and GC B cells was dramatically increased by the IM‐Gel (NP‐TLR7/8a) over two weeks after vaccination compared to NP‐TLR7/8a (Figure 4a). This led to significantly higher production of serum antibody and responses, which lasted for more than two months (Figure 4a).

Next, we analyzed B‐ and T‐cell immune responses after priming and booster vaccination at an interval of 2 weeks (Figure 4b). The absolute numbers of germinal center T_fh_ CD4 cells (CXCR5^+^ PD‐1^+^) and GC B cells were significantly increased by NP‐TLR7/8a (dose escalation) and IM‐Gel (NP‐TLR7/8a) compared with NP‐TLR7/8a (bolus) (Figure 4c; Figure S15, Supporting Information). Breadth of serum antibody responses were also significantly higher in NP‐TLR7/8a (dose escalation) and IM‐Gel (NP‐TLR7/8a) (Figure 4d). Notably, compared with alum‐vaccinated mice, IM‐Gel (NP‐TLR7/8a)‐vaccinated mice demonstrated better germinal center T_fh_ CD4 cells, GC B cells, and serum IgG titer responses. In addition to humoral responses, antigen‐specific CD8^+^ T cells were generated in the acute‐infection‐like vaccinated groups (NP‐TLR7/8a (dose escalation) and IM‐Gel (NP‐TLR7/8a) compared to the NP‐TLR7/8a vaccinated group (Figure 4e; Figure S16, Supporting Information). Furthermore, we evaluated the dose‐dependent immune responses of IM‐Gel (NP‐TLR7/8a + OVA) by assessing serum OVA‐specific IgG titers and the population of GC B cells (Figure S17, Supporting Information).

### 2.4 IM‐Gel (NP‐TLR7/8a) with sM2HA2 Ensures Cross‐Protection Against Heterologous Influenza Subtypes and Long‐Lasting Immune Responses

Because influenza viruses continuously undergo antigenic drift, resulting in new variants, it is crucial to develop cross‐reactive vaccines that provide broad protection across multiple viral variants to obtain a universal influenza vaccine. To enhance the broad protection provided by influenza M2 and HA2, we combined these antigens with an IM‐Gel (NP‐TLR7/8a) and evaluated their potential as candidates for universal influenza vaccines.

**Figure 5:**
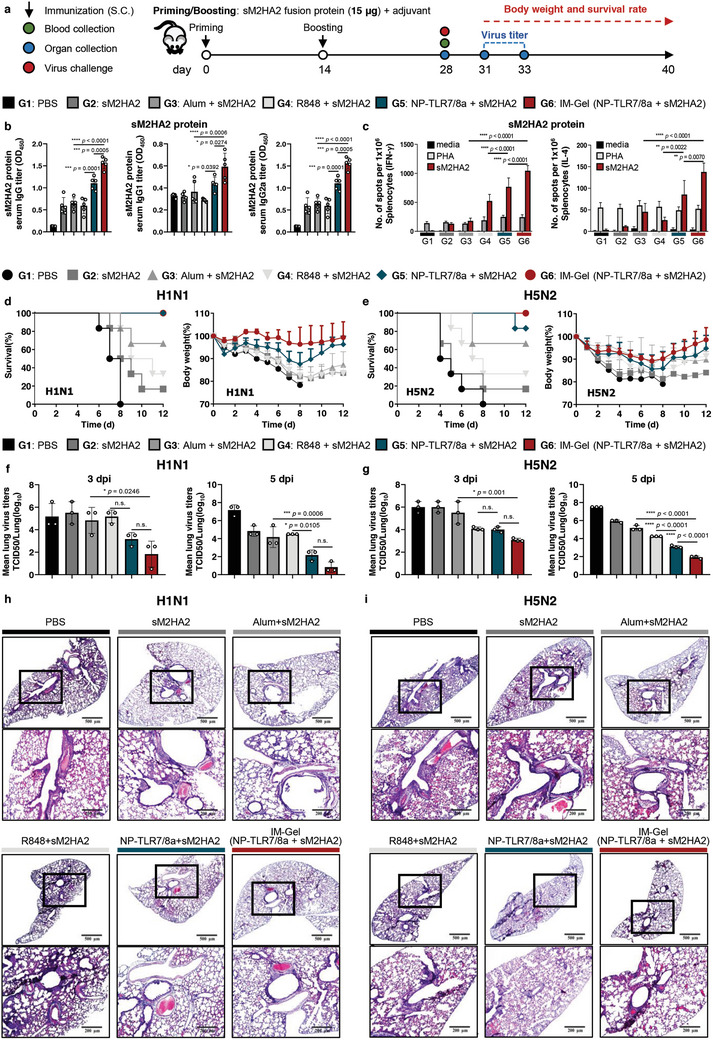
Generation of influenza virus‐specific B and T cells responses via IM‐Gel (NP‐TLR7/8a) with sM2HA2 and its cross‐protection against H1N1 and H5N2. a) Injection and analysis schedule and dose of sM2HA2 fusion protein vaccination with adjuvant. Indicated samples (sM2HA2 protein 15 µg, TLR7/8a 30 nmol) were injected two times with a 14‐day interval. b) Serum sM2HA2 protein‐specific IgG, IgG1, and IgG2a titer (*n* = 5). c) Number of spots of IFN‐γ‐ or IL‐4‐secreting splenocytes after sM2HA2 protein restimulation determined suing the ELISPOT assay (*n* = 4). d) Survival rate and body weight change after infection with H1N1 variant (*n* = 6). e) Survival rate and body weight change after infection with H5N2 variant (*n* = 6). f) Lung viral titers 3 or 5 days after infection with H1N1 variant (*n* = 3). g) Lung viral titers 3 or 5 days after infection with H5N2 variant (*n* = 3). h) Representative image of H&E staining of lung from immunized mice, demonstrating the inflammation responses after infection with H1N1 variant. i) Representative image of H&E staining of lung from immunized mice, demonstrating the inflammation responses after infection with H5N2 variant. All data are presented as the mean ± s.d. Statistical significance was evaluated by one‐way ANOVA with Tukey's multiple comparison test in b, c, f, and g. *p* values: NS, not significant; *p < 0.05, **p < 0.01, ***p < 0.001, ****p < 0.0001.

To validate whether the IM‐Gel (NP‐TLR/8a + sM2HA2) can induce robust antigen‐specific immune responses toward the sM2HA2 universal vaccine candidate, mice were subcutaneously vaccinated with the sM2HA2 antigen with an adjuvant, and antigen‐specific IgG titers and T cell immune responses were evaluated (**Figure** **5**a). Serum total IgG, IgG1, and IgG2a responses specific to sM2HA2 were all significantly higher in the group vaccinated with the IM‐Gel (NP‐TLR7/8a + sM2HA2) compared to those in the group receiving well‐known alum or other adjuvants (Figure 5b). Additionally, the M2‐ and HA2‐specific IgG levels were higher in the groups that received sM2HA2 with the IM‐Gel (NP‐TLR7/8a) than in the alum and other adjuvant groups (Figure S18, Supporting Information). Because optimal antiviral activity, including B cell activation, antibody isotype switching, and CD8^+^ T cell cytotoxicity, relies heavily on cognate signals and secreted factors from T cells, we investigated the effect of the IM‐Gel (NP‐TLR7/8a + sM2HA2) on T cell activation.^[^ ^24^ ^]^ Splenic lymphocytes of vaccinated mice were tested for their ability to secrete IFN‐γ and IL‐4 after stimulation with antigen or antigen‐specific peptides. Mice vaccinated with IM‐Gel (NP‐TLR7/8a + sM2HA2) showed increased numbers of antigen or peptide‐specific IFN‐γ and IL‐4‐secreting cell populations compared to those in other adjuvant groups (Figure 5c). Taken together, the IM‐Gel (NP‐TLR7/8a + sM2HA2) adjuvant effectively induced both humoral and cell‐mediated immunity.

To evaluate whether the sM2HA2‐induced immune response generates broad protection against lethal infection with multiple influenza viral strains, vaccinated mice were challenged with 10 LD_50_ of mouse‐adapted H1N1 or H5N2 influenza A subtypes. Protective efficacy was determined by survival rates and weight loss, which were monitored daily for 12 dpi (Figure 5a). In the analysis of lethal infection with H1N1 and H5N2, IM‐Gel (NP‐TLR7/8a + sM2HA2)‐immunized group demonstrated 100% survival with insignificant weight loss across mice (Figure 5d,e). Analysis of virus titers in the lungs of challenged mice at days 3 and 5 showed that compared with alum + sM2HA2, IM‐Gel (NP‐TLR7/8a + sM2HA2)‐immunized mice had significantly reduced viral loads upon lethal infections with H1N1 or H5N2 strains (Figure 5f,g). Viral clearance in the pathological lesions of lungs infected with the influenza virus, including edema, interstitial pneumonia, and infiltration of inflammatory cells around the bronchi and bronchioles, was further analyzed by H&E staining (Figure 5h,i). Inflammatory cell infiltration around the bronchi and bronchioles was detected in the control group after viral infection. However, the lungs from mice immunized with the IM‐Gel (NP‐TLR7/8a + sM2HA2) showed reduced inflammatory infiltration than the control groups.

**Figure 6:**
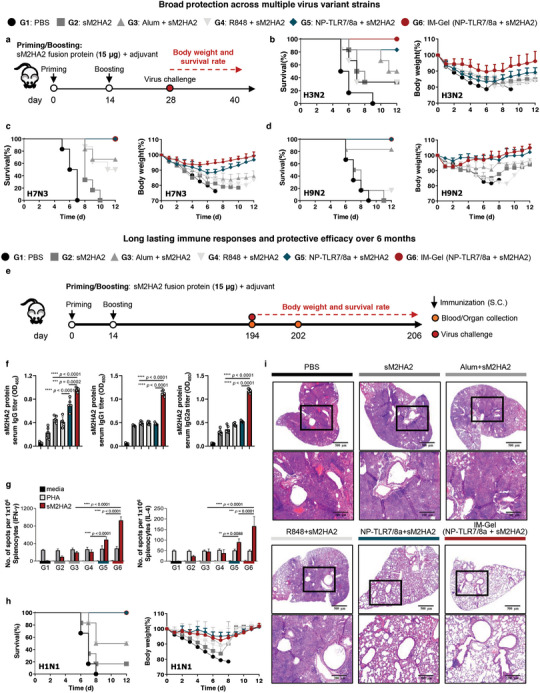
Cross‐protection against influenza subtypes and long‐lasting immune response over 6 months induced via IM‐Gel (NP‐TLR7/8a) with sM2HA2, as a universal influenza vaccine. a–d) Broad protection across multiple viral variants of influenza. Injection and analysis schedule and dose of sM2HA2 fusion protein vaccination with adjuvant. Indicated samples (sM2HA2 protein 15 µg, TLR7/8a 30 nmol) were injected two times with a 14‐day interval (a). Survival rate and body weight change after infection with b) H3N2 variant, c) H7N3 variant, and d) H9N2 variant (*n* = 6). e–i) Long lasting immune responses and protective efficacy over 6 months. Injection and analysis schedule and dose of sM2HA2 fusion protein vaccination with adjuvant. Indicated samples (sM2HA2 protein 15 µg, TLR7/8a 30 nmol) were injected two times with a 14‐day interval, and the virus challenge was conducted 6 months after the last vaccination (e). Serum sM2HA2 protein‐specific IgG, IgG1, and IgG2a titer (*n* = 5) (f). Number of spots of IFN‐γ‐ or IL‐4‐secreting splenocytes after sM2HA2 protein restimulation determined suing the ELISPOT assay (*n* = 5) (g). Survival rate and body weight change after infection with H1N1 variant (*n* = 6) (h). Representative image of H&E staining of lung from immunized mice, demonstrating the inflammation responses after infection with H1N1 variant (i). All data are presented as the mean ± s.d. Statistical significance was evaluated by one‐way ANOVA with Tukey's multiple comparison test in (f) and (g). *p* values: NS, not significant; *p < 0.05, **p < 0.01, ***p < 0.001, ****p < 0.0001.

To further evaluate the broad protection against lethal infection with multiple influenza virus strains, the vaccinated mice were challenged with 10 LD_50_ of mouse‐adapted H3N2, H7N3, or H9N2 influenza A subtypes (**Figure** **6**a). In the alum‐immunized group, only half of the mice survived lethal infection with H3N2, two‐thirds survived H7N3, and five‐sixths survived H9N2 infection, all of which were associated with significant weight loss. Interestingly, the IM‐Gel‐immunized group showed 100% survival against lethal infections with H3N2, H7N3, and H9N2 (Figure 6b–d).

The duration of protection conferred by a vaccine is a critical measure for assessing its long‐term effectiveness. To verify whether the spatiotemporal infection‐mimicking properties of the IM‐Gel could generate a long‐term protective effect, we immunized mice and evaluated their antigen‐specific humoral and cellular immune responses 6 months after boosting immunization (Figure 6e). The IM‐Gel (NP‐TLR7/8a + sM2HA2) showed significantly higher levels of sM2HA2‐specific IgG, IgG1, and IgG2a, as well as sM2 and HA2 peptide‐specific IgG than the other groups (Figure 6f; Figure S19, Supporting Information). Furthermore, cell‐mediated immune response, indicated by levels of IFN‐γ and IL‐4 in response to sM2HA2 protein, sM2 peptide, and HA2 peptide, were higher in the IM‐Gel (NP‐TLR7/8a + sM2HA2) group compared to other vaccine groups, which showed lower levels of IFN‐γ and IL‐4 (Figure 6g; Figure S19, Supporting Information).

Durable humoral and cellular immune responses generate long‐term protection, as evaluated by restrained viral replication after H1N1 infection. All mice immunized with the IM‐Gel (NP‐TLR7/8a + sM2HA2) survived a lethal dose of H1N1, whereas only half of the alum + sM2HA2 immunized mice survived (Figure 6h). To assess the inhibitory efficacy of viral replication and the severity of inflammation, we examined the lungs of mice infected with H1N1 at 7 dpi using H&E staining. The control group exhibited severe interstitial pneumonia induced by the infiltration of inflammatory cells, whereas the IM‐Gel (NP‐TLR7/8a + sM2HA2)‐immunized group showed only mild infiltration of inflammatory cells around the bronchioles (Figure 6i). Taken together, these results demonstrate that the IM‐Gel exhibits enhanced effectiveness in reducing both viral replication and inflammation following infection.

### 2.5 IM‐Gel (NP‐TLR7/8a) with SARS‐CoV‐2 Spike Protein Elicits Cross‐Reactive Antibody Response Toward Multiple SARS‐CoV‐2 Variants

COVID‐19, a disease attributed to the novel coronavirus SARS‐CoV‐2, was classified as a pandemic by the World Health Organization in March 2020. To cope with this unprecedented crisis, global scientific communities have intensified efforts to develop vaccine candidates, and several have achieved emergency authorization from the U.S. Food and Drug Administration (FDA).^[^ ^25^ ^]^ Various subunit vaccines, including messenger RNA, protein subunits, and adenovirus‐vectored vaccines, have been deployed worldwide.^[^ ^26^ ^]^ However, current FDA‐approved SARS‐CoV‐2 vaccines continue to show a reduced response rate to new variants of concern (VOCs), reflecting the challenges in maintaining vaccine efficacy as the virus evolves.^[^ ^27^ ^]^ Therefore, a vaccine platform that can provide greater breadth against SARS‐CoV‐2 VOCs is urgently needed. We used the SARS‐CoV‐2 spike protein with IM‐Gel strategy and evaluated its broader efficacy against SARS‐CoV‐2 VOCs. The spike protein of SARS‐CoV‐2 was chosen as the target antigen because it mediates viral entry by binding to the host receptor ACE2 via the receptor‐binding domain (RBD).^[^ ^28^ ^]^

**Figure 7:**
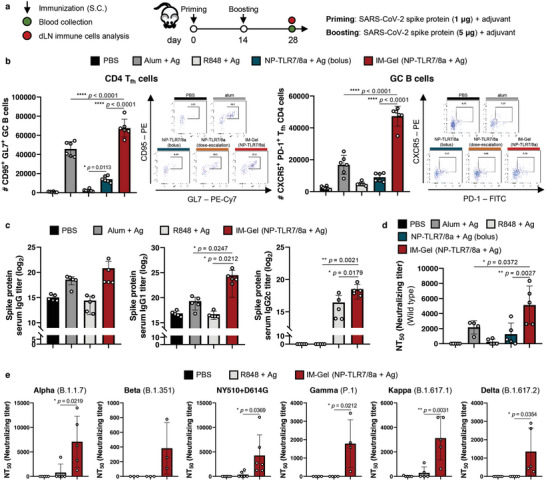
Cross‐reactive antibody response toward multiple variants of SARS‐CoV‐2. a) Injection and analysis schedule and dose of SARS‐CoV‐2 spike protein vaccination with adjuvant. Indicated samples (SARS‐CoV‐2 spike protein 1 µg for priming and 5 µg for boosting, TLR7/8a 30 nmol) were injected two times with a 14‐day interval. Blood and dLN were collected for immune analysis 14 days after boosting. b) Number of GC B cells (CD95^+^ GL7^+^ B220^+^ IgD^−^ cells) and CD4^+^ T_fh_ cells (CXCR5^+^ PD‐1^+^ CD4^+^ cells) in dLN (*n* = 6). c) Serum SARS‐CoV‐2 spike protein‐specific IgG, IgG1 and IgG2c titer (*n* = 5). d) Neutralizing titer against SARS‐CoV‐2 variants via pseudovirus‐based neutralizing assay (*n* = 4 for Alum + Ag; *n* = 5 for the others). All data are presented as the mean ± s.d. Statistical significance was evaluated by one‐way ANOVA with Tukey's multiple comparison test in (b–e). *p* values: NS, not significant; *p < 0.05, **p < 0.01, ***p < 0.001, ****p < 0.0001.

Prime‐boosting vaccination of the SARS‐CoV‐2 spike protein with IM‐Gel (NP‐TLR7/8a) effectively generated CD4 T_fh_ cells and GC B cells in the dLNs (**Figure** **7**a,b), as well as high serum titers of anti‐spike IgGs (Figure 7c). To determine whether the IM‐Gel (NP‐TLR7/8a) could enhance the breadth of antibody responses against SARS‐CoV‐2 variants, we characterized the neutralizing antibody titer against several VOCs. Compared to the wild‐type, the IM‐Gel (TLR7/8a)‐adjuvanted spike protein vaccinated group produced the highest neutralizing antibody titer, significantly surpassing that of the FDA‐approved alum‐adjuvanted group. For other variants, the IM‐Gel (TLR7/8a)‐adjuvanted spike protein protein‐vaccinated group developed high neutralizing antibody titers against heterologous variants (Alpha, Beta, NY510+D614G, Gamma, Kappa, and Delta), a response that was not observed in the R848‐adjuvanted group.

## 3 Conclusion

In the present study, we developed a novel next‐generation biomaterial‐based vaccine platform that mimics acute viral infections. The innovative IM‐Gel formulation, achieved through physical cross‐linking between NP‐TLR7/8a and TA, demonstrated superior bioadhesiveness and controlled spatiotemporal release, maintaining a stable presence at the injection site and in dLNs, over several days. This spatiotemporal control of antigen and adjuvant delivery effectively mimicked acute infection‐like conditions, since the continuous presentation of antigen and adjuvant signals over 7 days and deep intra‐LN penetration via the conduit led to enhanced antigen‐specific humoral and cellular immune responses (Figure 1c,d). Notably, CD8^+^ T cells were observed to play a leading role in fighting acute virus infections (Figure S20, Supporting Information).

We further validated the potential of the IM‐Gel (NP‐TLR7/8a) for the development of universal influenza and broad‐spectrum SARS‐CoV‐2 vaccines. The combination of sM2HA2 antigens conferred cross‐protection against multiple influenza subtypes (H1N1, H5N2, H3N2, H7N3, and H9N2) with significant survival rates and reduced viral loads in lethal infection models. It also demonstrated long‐term immune responses, which showed a significant effect 6 months after infection. Additionally, the application of the SARS‐CoV‐2 spike protein with the IM‐Gel (NP‐TLR7/8a) elicited strong cross‐reactive antibody responses against multiple SARS‐CoV‐2 variants (Alpha, Beta, NY510+D614G, Gamma, Kappa, and Delta) of concern, highlighting the platform's ability to address challenges posed by viral mutations (Figure 1e).

Furthermore, we compared the novel IM‐Gel vaccine platform with existing vaccine platforms, such as mRNA lipid nanoparticle (LNP) vaccines (Figure S21, Supporting Information) and alum‐based protein vaccines (Figures 4, 5, and 6a‐i). IM‐Gel demonstrated superior vaccination efficacy, underscoring the necessity of a spatiotemporal dynamic immunization strategy for next‐generation vaccines.

In summary, the IM‐Gel (NP‐TLR7/8a) system is a promising platform for vaccine development that offers controlled intra‐LN delivery, sustained immune activation, and broad protection against diverse pathogens. This strategy has the potential to significantly improve the efficacy and durability of vaccines, particularly against rapidly evolving infectious diseases.

## 4 Experimental Section

### Synthesis of Nanoparticulate‐TLR7/8a (NP‐TLR7/8a)

Transiently masked TLR7/8a was synthesized using the scheme shown in Figures S1–S4 (Supporting Information). The structures of the synthesized compounds were characterized using liquid chromatography–mass spectrometry (LC‒MS; Agilent 1260–6120 system) and high‐performance liquid chromatography (HPLC; Waters Acquity UPLC H‐Class instrument). To synthesize NP‐TLR7/8a, recombinant mouse serum albumin (MSA) (Albumin Bioscience) in PBS (10 mg mL^−1^) was reacted with 15 equivalents of 3‐Mercaptopropionic acid NHS ester (MedChemExpress) (20 mg mL^−1^) in dimethyl sulfoxide (DMSO) for 1 h at room temperature (RT) to thiolate MSA. The resultant product was purified by desalting (Zeba spin desalting columns, Thermo Fisher Scientific) twice to remove the unreacted 3‐Mercaptopropionic acid NHS ester. Thiolated MSA was then reacted with 25 equivalents of transiently masked TLR7/8a (15 mg mL^−1^) in DMSO. The conjugation reaction was performed at 4 °C for 1 h. The resulting immunoconjugates were purified by desalting twice to remove unreacted transiently masked TLR7/8a. To synthesize fluorescent NP‐TLR7/8a, NP‐TLR7/8a (50 mg mL^−1^) and sulfo‐Cyanine5 NHS ester (10 mg mL^−1^) were dissolved in 0.1 m sodium bicarbonate (pH 8.3). Sulfo‐Cyanine5 NHS ester was added to NP‐TLR7/8a and allowed to react for 1 h in the dark. The mixture was filtered twice through the Pierce dye removal columns (Thermo Fisher Scientific) to remove unlabeled free dye.

### Characterization of NP‐TLR7/8a

The loaded, transiently masked TLR7/8a was quantified using ultraviolet‐visible light spectrometry (UV‐1800). The hydrodynamic size was determined by dynamic light scattering (DLS) using an ELS‐Z electrophoretic light scattering photometer. Morphological analyses were performed using a field‐emission scanning electron microscope (JSM‐7000F).

### Synthesis of IM‐Gel (NP‐TLR7/8a)

A solution of NP‐TLR7/8a (3.2 mg mL^−1^, PBS) was mixed vigorously with tannic acid (TA) (8.5 mg mL^−1^, PBS) at a 1:1 volume ratio to achieve a stoichiometric ratio of [TA]/[NP‐TLR7/8a] of 2.66. The mixture was incubated at room temperature for 30 min to induce coacervate formation. The mixture was centrifuged at 1500 × g for 3 min; this process was repeated twice to effectively remove any excess TA. The fluorescent IM‐Gel (NP‐TLR7/8a) was prepared similarly, except that fluorescently labeled NP‐TLR7/8a was used instead of regular NP‐TLR7/8a.

### Characterization of IM‐Gel (NP‐TLR7/8a)

The turbidity of the solution was assessed using UV–vis spectroscopy by measuring absorbance at 600 nm. Hydrodynamic size was determined using dynamic light scattering (DLS). Morphological analysis was performed using field‐emission scanning electron microscopy. The rheological properties of the BIND were analyzed using an ARES‐G2 rheometer (TA Instruments). All measurements were conducted at 25 °C using a parallel steel plate geometry with a diameter of 25 mm. Initially, viscosity was assessed under increasing shear force through a stepped flow test (0.1–100 s⁻¹, 1 Hz frequency). Subsequently, the resilience and fluid strength after the application of a strong strain were evaluated. The storage modulus (G′) and loss modulus (G″) were measured using oscillatory time sweep experiments for 3 min (0.2% strain, 1 Hz frequency) prior to applying a strong strain (1 min, 500% strain, 1 Hz frequency). Finally, the oscillatory time sweep mode was resumed (2 min, 0.2% strain, 1 Hz frequency). To assess the bioadhesiveness, a 150 µL sample was applied at the interface of two pieces of porcine skin, and a shear stress–strain curve was generated using a universal testing machine (UTM, Instron 5943). The adhesion energy (Gad) for each sample was calculated using the equation: (*G_ad_* =  3(*F*/*w*)^2^/(2*Eh*)), where F is the measured adhesive tensile stress at the maximum force, w and h are the width (30 mm) and height (20 mm) of the porcine skin, respectively, and E is the tensile modulus of the porcine skin.

### Safety Statement

No unexpected or unusually high safety hazards were encountered.

### In Vitro Bone Marrow‐Derived Dendritic Cell (BMDC) Culture and Cytokine Production

The femurs and tibias of C57BL/6 mice (6‐ to 8‐week‐old females) were isolated, and the bone marrow was extracted by flushing with RPMI 1640 medium (without HEPES, Thermo Fisher Scientific) using a 26‐gauge syringe. Red blood cells (RBCs) were lysed and removed using RBC lysis buffer (BioLegend). After washing, the cells were resuspended in RPMI medium containing mGM‐CSF (20 ng mL^−1^, CreaGene) and seeded (2.5 × 10^6^ per well) in a 6‐well culture plate. On day 2, the medium containing mGM‐CSF (20 ng mL^−1^) was refreshed after thorough washing with PBS to remove the non‐adherent cells. On day 4, a fresh medium containing mGM‐CSF (20 ng mL^−1^) was added. On day 6, the differentiated immature BMDCs were treated with the indicated samples and incubated for 36 h. Cell supernatants were collected and the concentrations of cytokines (IL‐12(p70), IL‐6, TNF‐α, and IL‐10) were analyzed using the OptEIA enzyme‐linked immunosorbent assay (ELISA) kit (BD Science) according to the manufacturer's instructions.

### Animals and Antibodies

All animal experiments were performed in strict compliance with the National Institutes of Health's (NIH) Guide for the Care and Use of Laboratory Animals. The Institutional Animal Care and Use Committee (IACUC) of Chungnam National University (approval number: 202306A‐CNU‐098) and IACUC of the Sungkyunkwan University School of Medicine (approval number: SKKUIACUC2020‐12‐13‐1) approved all procedures in mice. All efforts were made to minimize animal suffering. C57BL/6 mice (6‐ to 8‐week‐old females) were purchased from Orient Bio (Korea). BALB/c mice (5‐week‐old females) were purchased from the Hanil Laboratory Animal Center (Jeonju, Korea). All animals were housed individually, in ventilated cages under 30–70% humidity at 21–26 °C on a 12 h light‐dark cycle. Detailed information on the antibodies used in this study, such as the fluorescent antibody type, manufacturer, clone, and catalog number, is provided in Table S1 (Supporting Information).

### In Vivo Fluorescence Imaging

Fluorescence at the injection site or in the LN was measured by analyzing whole mice or LNs collected from animals using an optical imaging IVIS Lumina XR in vivo imaging system (Perkin Elmer).

### Collagen Binding Analysis with SpongeCol

To assess the collagen binding ability, NP‐TLR7/8a‐Cy5 or IM‐Gel (NP‐TLR7/8a‐Cy5) was added to commercial type I collagen‐based porous scaffold SpongeCol (Advanced Biomatrix) and incubated for 1 h at 37 °C. After incubation, the SpongeCol was vigorously washed with PBS 10 times, and fluorescence images were obtained with a Leica TCS SP8 confocal laser scanning microscope.

### Cryo‐Section and Immunofluorescence Imaging

The extracted lymph nodes (LNs) were immersed in 4% paraformaldehyde for 24 h and then equilibrated in a 30% sucrose solution for another 24 h. The sections were subsequently embedded in Tissue‐Tek OCT compound (Sakura). After freezing, LNs were sectioned using a Leica rotary microtome (RM2165). The tissue sections were washed and blocked with a 5% FBS solution for 1 h. After washing with PBS, the sections were stained with antibodies overnight at 4 °C. Detailed information on the antibodies used is provided in Table S1 (Supporting Information). Fluorescence images were captured using a Leica TCS SP8 confocal laser scanning microscope.

### In Vivo Single Cells Preparation and Flow Cytometry

To prepare single cells from tissue, inguinal lymph nodes (LNs) were mechanically disrupted and resuspended in a medium containing collagenase D (1 mg mL^−1^, Sigma‒Aldrich). The cell suspension was incubated in a shaking incubator for 40 min at 37 °C. Following incubation, the cells were filtered through 70‐µm cell strainers and washed twice with PBS. For the analysis of CD4^+^ Tfh and GC B cells, single cells were stained with BD Horizon Fixable Viability Stain 780 and antibodies specific to the surface markers of CD4^+^ Tfh cells (anti‐mouse CD4, PD‐1, and CXCR5) and GC B cells (anti‐mouse B220, IgD, GL‐7, and FAS). Detailed antibody information is available in Table S1 (Supporting Information) and the gating strategies used are shown in Figure S15 (Supporting Information).

For antigen‐specific CD8^+^ T‐cell analysis, single cells (5 × 10^5^ per well) were seeded in a round‐bottom 96‐well plate and restimulated with the OVA peptide SIINFEKL (10 µg mL^−1^) and GolgiPlug (a protein transport inhibitor, 0.6 µg mL^−1^, BD Bioscience) for 6 h. The cells were then stained with BD Horizon Fixable Viability Stain 780 and surface marker antibodies for CD8^+^ T cells (anti‐mouse CD3 and CD8) for 30 min at 4 °C. Detailed antibody information is presented in Table S1 (Supporting Information). For intracellular staining, the cells were fixed and permeabilized with fixation/permeabilization solution for 20 min at 4 °C. Fixed cells were washed twice with BD Perm/Wash buffer (BD Bioscience) and stained with antibodies specific to cytokine‐producing CD8^+^ T cells (anti‐mouse IFN‐γ, TNF‐α, and granzyme B) for 30 min at 4 °C. After staining, the cells were washed twice with BD Perm/Wash buffer and resuspended in the staining buffer. Flow cytometry data were analyzed using a BD FACSCanto II (The BIORP of the Korea Basic Science Institute (KBSI) and quantified using FlowJo V10. Detailed antibody information is presented in Table S1 (Supporting Information) and the gating strategies used are shown in Figure S16 (Supporting Information).

### Preparation of Recombinant sM2HA2 Fusion Protein

The recombinant sM2HA2 fusion protein was prepared as described previously.^[^ ^18^ ^]^ Briefly, *Escherichia coli* BL21 (DE3) cells were transformed with the plasmid pRSET‐A–sM2HA2 using the heat shock method. The resulting colonies were seeded in 5 ml LB broth supplemented with 100 mg mL^−1^ ampicillin and incubated at 37 °C with shaking. Subsequently, the culture was scaled up to a larger volume of LB medium under the same conditions. When the optical density at 600 nm (OD_600_) reached 0.6, 1 mm isopropyl‐β‐D‐thiogalactopyranoside was added to induce the expression of the target protein. The cell pellets were collected, lysed, and protein was purified.

### In Vivo Antibody Titer Analysis

Serum was isolated from blood samples by centrifugation at 10000 × g for 20 min, and an ELISA was used to measure serum IgG levels. Briefly, antigen (200 ng per well of OVA, sM2HA2 recombinant protein, sM2 peptide or HA2 peptide) were coated onto 96‐well ELISA plates and incubated overnight at 4 °C. The plates were then blocked with 10% skim milk. Subsequently, serum dilution series and diluted horseradish peroxidase‐conjugated goat anti‐mouse IgG (GeneTex), IgG1, or IgG2a (Invitrogen) were added sequentially with relevant washing steps using PBS containing 0.05% Tween 20 (PBST). Finally, a tetramethylbenzidine (TMB, Millipore) substrate solution was added to initiate the reaction, and after 10 min, a stop solution (2N H_2_SO_4_) was added. The absorbance was measured at 450 nm using an ELISA auto‐reader (Molecular Devices). The detailed peptide sequence information is provided in Table S2 (Supporting Information).

### Antigen‐Specific T Cell Response Analysis with Enzyme‐Linked Immunospot (ELISPOT)

As previously described,^[^ ^2^ ^]^ mouse IFN‐γ and IL‐4 ELISPOT kits (BD Biosciences) were used to assess antigen‐specific T cell responses. Briefly, isolated splenocytes (1 × 10^6^ cells per well) were cultured in BD ELISPOT 96‐well plates that had been coated with anti‐mouse IFN‐γ and IL‐4 capture antibodies (5 µg mL^−1^) overnight at 4 °C. The splenocytes were then aseptically extracted and stimulated with either sM2HA2 recombinant protein, sM2 or HA2 peptide (1 µg per well), medium alone (negative control) or 0.5 µg mL^−1^ phytohemagglutinin (positive control; Invitrogen). Following a 24 h incubation at 37 °C with 5% CO_2_, the plates were treated with streptavidin‐horseradish peroxidase, biotinylated anti‐mouse IFN‐γ and IL‐4 antibodies, and substrate solution (BD Bioscience), according to the manufacturer's protocol. Finally, the spots were counted using an ImmunoScan Entry Analyzer (Cellular Technology, Shaker Heights).

### Viruses and Challenge Experiments

All mouse studies involving the virus were conducted at biosafety level (BSL)‐2 facilities. To evaluate the protective efficacy and compare the performance of the sM2HA2 vaccine adjuvanted with NP‐TLR7/8a or IM‐Gel against diverse influenza virus subtypes, mice were intranasally infected (i.n.) with tenfold the mouse LD_50_ mouse‐adapted low‐pathogenic AI A/Puerto Rico/8/34(H1N1), A/aquatic bird/Korea/W81/2005(H5N2), A/aquatic bird/Korea/W44/2005(H7N3), A/Chicken/Korea/116/2004 (H9N2), and A/Philippines/2/2008 (H3N2). All the divergent viruses used in the challenge experiments were kindly provided by Dr. Young Ki Choi (College of Medicine and Medical Research Institute, Chungbuk National University, Cheongju, Republic of Korea). Survival and weight changes in the mice were monitored at consistent intervals for 12 days. Survival rate was indicated by 20% loss of body weight post‐challenge, at which point animals were euthanized. Six mice from each group were euthanized at 3‐ and 5‐days post‐infection (dpi) to evaluate viral titers in lungs. All attempts were made to reduce suffering, and after the final monitoring, CO_2_ inhalation was used to humanely euthanize all surviving mice.

### Viral Titers and Histopathological Analysis of Lungs

As previously described,^[^ ^29^ ^]^ the collected lung tissues were homogenized in PBS containing an antibiotic and antimycotic solution (Gibco), and then centrifuged at 12 000 × g to purify the viral extract. The extracted virus was then added to confluent MDCK in ten‐fold serial dilutions and incubated for one hour at 37 °C. After the initial incubation, the infected cells were incubated for 3 days in a medium containing L‐1‐tosylamide‐2‐phenylethyl chloromethyl ketone (TPCK) trypsin (Sigma‒Aldrich). Hemagglutination (HA) assay was performed to determine cytopathic effects (CPE). Viral titers were calculated using the Reed and Muench method and are shown as log_10_ TCID_50_/lung sample. For histopathological analysis, collected lung tissues were immersed in 10% neutralized buffered formalin (NBF) for two days. The middle of the lobe was embedded in paraffin wax and placed on slide glass as 4–6 µm thickness. The slides were stained with hematoxylin and eosin (H&E) method.

### Production of SARS‐CoV‐2 Spike Pseudovirus

SARS‐CoV‐2 spike mutants were generated by site‐directed mutagenesis or artificially synthesized DNA from the SARS‐CoV‐2 mutant spikes. The SARS‐CoV‐2 pseudovirus was produced by co‐transfection of 293T cells with pMDLg/pRRE (Addgene plasmid, 12251), pRSV‐Rev (Addgene plasmid, 12253), pCDH‐CMV‐Nluc‐copGFP‐Puro (Addgene plasmid, 73037), and plasmids encoding the SARS‐CoV‐2 spike (pCMV3‐SARS‐CoV‐2 Spike, Sino Biological) using polyetherimide. Sixty hours post transfection, SARS‐CoV‐2 spike pseudoviruses containing culture supernatants were harvested, filtered (0.45 µm pore size, Millipore); the copy number of pseudoviruses was quantitated by qPCR (Takara Bio).

### Pseudovirus Neutralization Assay

Derivative 293T cells expressing angiotensin converting enzyme 2 (ACE2) were generated by transfecting 293T cells with ACE2 expression lentiviral vector (Addgene Plasmid, 145839). The cells were used as single‐cell clones derived by limiting dilution from the bulk populations. 293T‐ACE2 cells were seeded at a density of 2 × 10^4^ cells per well in 96‐well luminometer‐compatible tissue culture plates, 24 h before infection. For the neutralization assay, pseudoviruses (1.8 × 10^7^ copies) were mixed with diluted serum ranging from 50‐ to 984150‐fold and added to 96‐well 293T‐ACE2 cells. After 24 h of incubation, the inoculum was replaced with a fresh medium. Luciferase activity was measured 72 h after infection. Briefly, cells were lysed with 40 µL per well of Passive Lysis buffer (Promega). Luciferase activity in the lysates was measured using a Nano‐Glo Luciferase Assay System (Promega). Specifically, 40 µL of substrate in Nano‐Glo buffer was mixed with 40 µL cell lysate and incubated for 3 min at room temperature. NanoLuc luciferase activity was measured using a GloMax Navigator Microplate Luminometer (Promega) using 300 ms integration time. NT_50_ values were calculated by non‐linear regression, that is, log (inhibitor) versus response (three parameters), using GraphPad Prism 9.

### Synthesis of OVA mRNA‐Loaded Lipid Nanoparticle (LNP) Vaccine

OVA mRNA‐loaded lipid nanoparticle (LNP) vaccine were prepared using microfluidic device (NanoAssemblr Ignite, Precision Nanosystems). The organic phase was prepared by dissolving 100 mg mL^−1^ ALC‐0315 (MedChemExpress), 50 mg mL^−1^ ALC‐0159 (MedChemExpress), 10 mg mL^−1^ 1,2‐distearoyl‐sn‐glycero‐3‐phosphocholine (DSPC) (Avanti), and 10 mg mL^−1^ cholesterol (Sigma‒Aldrich) in ethanol at a weight ratio of 43/5/9/20. Meanwhile, the aqueous phase contained 0.1 mg mL^−1^ CleanCap OVA mRNA dissolved in pH 4 buffer (100 mm sodium citrate). The organic and aqueous phases were mixed at a 3:1 volume ratio using a microfluidic device. The synthesized LNPs were purified by overnight dialysis in 1X PBS using Slide‐A‐Lyzer Dialysis Cassettes, 10K MWCO (Thermo Fisher).

### Antibody‐Mediated Depletion of Immune Cells in Vaccinated Mice

Experiments were conducted as previous papers described.^[^ ^30^, ^31^ ^]^ Briefly, mice were administered monoclonal antibodies via intraperitoneal injection for depleting CD20 T cells or CD8^+^ T cells at a dose of 100 µg in PBS on days −4, −2, 0, +3, and +5 following the influenza challenge. Survival ratio and body weight were examined.

### Statistical Analysis

All results are indicated as the mean ± s.d. A two‐tailed unpaired t‐test was used to compare the two groups. One‐way analysis of variance (ANOVA) with Tukey's multiple comparisons test was used to analyze multiple groups of data. All statistical analyses were performed using GraphPad Prism 8 and Microsoft Excel 2016. P values (NS, not significant, *p < 0.05, **p < 0.01, ***p < 0.001, and ****p < 0.0001) were used to indicate statistical significance.

## Conflict of Interest

The authors declare no conflict of interest.

## Supporting information



Supporting Information

## Data Availability

The data that support the findings of this study are available from the corresponding author upon reasonable request.

## References

[1] A. J. Pollard , E. M. Bijker , Nat. Rev. Immunol. 2021, 21, 83. 33353987 10.1038/s41577-020-00479-7 PMC7754704

[2] C. J. Wei , M. C. Crank , J. Shiver , B. S. Graham , J. R. Mascola , G. J. Nabel , Nat. Rev. Drug. Discov. 2020, 19, 239. 32060419 10.1038/s41573-019-0056-x PMC7223957

[3] P. S. Arunachalam , A. C. Walls , N. Golden , C. Atyeo , S. Fischinger , C. Li , P. Aye , M. J. Navarro , L. Lai , V. V. Edara , K. Röltgen , K. Rogers , L. Shirreff , D. E. Ferrell , S. Wrenn , D. Pettie , J. C. Kraft , M. C. Miranda , E. Kepl , C. Sydeman , N. Brunette , M. Murphy , B. Fiala , L. Carter , A. G. White , M. Trisal , C. L. Hsieh , K. Russell‐Lodrigue , C. Monjure , J. Dufour , et al., Nature 2021, 594, 253. 33873199 10.1038/s41586-021-03530-2

[4] C. Stein , H. Nassereldine , R. J. D. Sorensen , J. O. Amlag , C. Bisignano , S. Byrne , E. Castro , K. Coberly , J. K. Collins , J. Dalos , F. Daoud , A. Deen , E. Gakidou , J. R. Giles , E. N. Hulland , B. M. Huntley , K. E. Kinzel , R. Lozano , A. H. Mokdad , T. Pham , D. M. Pigott , R. C. Reiner , T. Vos , S. I. Hay , C. J. L. Murray , S. S. Lim , Lancet 2023, 401, 833. 36930674 10.1016/S0140-6736(22)02465-5 PMC9998097

[5] B. P. , E. A. A. Gillie , A. Roth , V. C. T. M. Picece , B. S. Ou , W. Luo , Nat. Rev. Mater. 2022, 7, 174. 34603749 10.1038/s41578-021-00372-2 PMC8477997

[6] A. L. Rasmussen , S. V. Popescu , Science 2021, 371, 1206. 33737476 10.1126/science.abf9569

[7] G. V. Reynoso , A. S. Weisberg , J. P. Shannon , D. T. McManus , L. Shores , J. L. Americo , R. V. Stan , J. W. Yewdell , H. D. Hickman , Nat. Immunol. 2019, 20, 602. 30886418 10.1038/s41590-019-0342-0 PMC6474694

[8] A. Schudel , D. M. Francis , S. N. Thomas , Nat. Rev. Mater. 2019, 4, 415. 32523780 10.1038/s41578-019-0110-7 PMC7286627

[9] T. H. O. Nguyen , L. C. Rowntree , B. Y. Chua , R. S. Thwaites , K. Kedzierska , Nat. Rev. Immunol. 2024, 24, 720. 38698083 10.1038/s41577-024-01029-1

[10] M. Sixt , N. Kanazawa , M. Selg , T. Samson , G. Roos , D. P. Reinhardt , R. Pabst , M. B. Lutz , L. Sorokin , Immunity 2005, 22, 19. 15664156 10.1016/j.immuni.2004.11.013

[11] S. E. Acton , L. Onder , M. Novkovic , V. G. Martinez , B. Ludewig , Trends Immunol. 2021, 42, 782. 34362676 10.1016/j.it.2021.07.003

[12] M. Shin , H. A. Lee , M. Lee , Y. Shin , J. J. Song , S. W. Kang , D. H. Nam , E. J. Jeon , M. Cho , M. Do , S. Park , M. S. Lee , J. H. Jang , S. W. Cho , K. S. Kim , H. Lee , Nat. Biomed. Eng. 2018, 2, 304. 30936449 10.1038/s41551-018-0227-9

[13] C. Chen , H. Yang , X. Yang , Q. Ma , RSC Adv. 2022, 12, 7689. 35424749 10.1039/d1ra07657d PMC8982347

[14] P. Sarker , P. K. Jani , L. C. Hsiao , O. J. Rojas , S. A. Khan , J. Colloid Interface Sci. 2023, 650, 541. 37423181 10.1016/j.jcis.2023.06.209

[15] P. Velmurugan , E. R. A. Singam , R. R. Jonnalagadda , V. Subramanian , Biopolymers 2014, 101, 471. 23996786 10.1002/bip.22405

[16] T. R. Mosmann , A. J. McMichael , A. LeVert , J. W. McCauley , J. W. Almond , Nat. Rev. Immunol. 2024, 24, 736. 38698082 10.1038/s41577-024-01030-8

[17] P. V. Markov , M. Ghafari , M. Beer , K. Lythgoe , P. Simmonds , N. I. Stilianakis , A. Katzourakis , Nat. Rev. Microbiol. 2023, 21, 361. 37020110 10.1038/s41579-023-00878-2

[18] H. J. Noh , M. Y. E. Chowdhury , S. Cho , J.‐H. Kim , H. S. Park , C.‐J. Kim , H. Poo , M.‐H. Sung , J.‐S. Lee , Y. T. Lim , J. Immunol. 2015, 195, 2472. 26216889 10.4049/jimmunol.1500492

[19] J. S. Tregoning , R. F. Russell , E. Kinnear , Hum. Vaccin. Immunother. 2018, 14, 550. 29232151 10.1080/21645515.2017.1415684 PMC5861793

[20] S. M. Jin , Y. J. Yoo , H. S. Shin , S. Kim , S. N. Lee , C. H. Lee , H. Kim , J. E. Kim , Y. S. Bae , J. H. Hong , Y. W. Noh , Y. T. Lim , Nat. Nanotechnol. 2023, 18, 390. 36635335 10.1038/s41565-022-01296-w

[21] H. H. Tam , M. B. Melo , M. Kang , J. M. Pelet , V. M. Ruda , M. H. Foley , J. K. Hu , S. Kumari , J. Crampton , A. D. Baldeon , R. W. Sanders , J. P. Moore , S. Crotty , R. Langer , D. G. Anderson , A. K. Chakraborty , D. J. Irvine , Proc. Natl. Acad. Sci. USA 2016, 113, E6639. 27702895 10.1073/pnas.1606050113 PMC5086995

[22] J. L. Turley , E. C. Lavelle , Curr. Opin. Immunol. 2022, 77, 102229. 35779364 10.1016/j.coi.2022.102229

[23] R. Roozendaal , T. R. Mempel , L. A. Pitcher , S. F. Gonzalez , A. Verschoor , R. E. Mebius , U. H. von Andrian , M. C. Carroll , Immunity 2009, 30, 264. 19185517 10.1016/j.immuni.2008.12.014 PMC2699624

[24] A. Sundararajan , L. Huan , K. A. Richards , G. Marcelin , S. Alam , H. M. Joo , H. Yang , R. J. Webby , D. J. Topham , A. J. Sant , M. Y. Sangster , PLoS One 2012, 7, 34377. 10.1371/journal.pone.0034377 PMC3311631 22457834

[25] X. Huang , E. Kon , X. Han , X. Zhang , N. Kong , M. J. Mitchell , D. Peer , W. Tao , Nat. Nanotechnol. 2022, 17, 1027. 35982317 10.1038/s41565-022-01174-5

[26] M. Sadarangani , A. Marchant , T. R. Kollmann , Nat. Rev. Immunol. 2021, 21, 475. 34211186 10.1038/s41577-021-00578-z PMC8246128

[27] W. Y. Chi , Y. Der Li , H. C. Huang , T. E. H. Chan , S. Y. Chow , J. H. Su , L. Ferrall , C. F. Hung , T. C. Wu , J. Biomed. Sci. 2022, 29, 34377. 10.1186/s12929-022-00853-8 PMC9569411 36243868

[28] C.‐L. Hsieh , J. A. Goldsmith , J. M. Schaub , A. M. Divenere , H.‐C. Kuo , K. Javanmardi , K. C. Le , D. Wrapp , A. G. Lee , Y. Liu , C.‐W. Chou , P. O. Byrne , C. K. Hjorth , N. V. Johnson , J. Ludes‐Meyers , A. W. Nguyen , J. Park , N. Wang , D. Amengor , J. J. Lavinder , G. C. Ippolito , J. A. Maynard , I. J. Finkelstein , J. S. Mclellan , Science 2020, 369, 1501. 32703906 10.1126/science.abd0826 PMC7402631

[29] E. S. Lee , Y. J. Shim , W. A. G. Chathuranga , Y. H. Ahn , I. J. Yoon , S. S. Yoo , J. S. Lee , Front. Vet. Sci. 2021, 8. 10.3389/fvets.2021.730700 PMC8677964 34926633

[30] M. B. Bull , H. Gu , F. N. L. Ma , L. P. Perera , L. L. M. Poon , S. A. Valkenburg , Sci. Adv. 2022, 8, eabl5209. 35385318 10.1126/sciadv.abl5209 PMC8986104

[31] J. Liu , J. Yu , K. McMahan , C. Jacob‐Dolan , X. He , V. Giffin , C. Wu , M. Sciacca , O. Powers , F. Nampanya , J. Miller , M. Lifton , D. Hope , K. Hall , N. P. Hachmann , B. Chung , T. Anioke , W. Li , J. Muench , A. Gamblin , M. Boursiquot , A. Cook , M. G. Lewis , H. Andersen , D. H. Barouch , Sci. Immunol. 2022, 7, eabq7647. 35943359 10.1126/sciimmunol.abq7647 PMC9407944

