# Supplementary material for: Spatiotemporal Dynamic Immunomodulation by Infection‐Mimicking Gels Enhances Broad and Durable Protective Immunity Against Heterologous Viruses
Source: Adv Sci (Weinh). 2025 Jan 13;12(9):2412116. doi: 10.1002/advs.202412116 (PMC11884557; doi:10.1002/advs.202412116)
Supplement: Supplementary file 1 — Supporting Information [file ADVS-12-2412116-s001.pdf]

ADVANCED SCIENCE
Open Access

Supporting Information

for *Adv. Sci.*, DOI 10.1002/advs.202412116

Spatiotemporal Dynamic Immunomodulation by Infection-Mimicking Gels Enhances Broad and Durable Protective Immunity Against Heterologous Viruses

Seung Mo Jin, Ju Hee Cho, Yebin Seong, Wijesinghe Arachchilage Gayan Chathuranga, Yejin Gwak, Young-Woock Noh, Min-Ho Lee, Sang-Seok Oh, Jin-Ho Choi, Jong-Soo Lee and Yong Taik Lim**

Supporting Information

Spatiotemporal Dynamic Immunomodulation by Infection-Mimicking Gels Enhances Broad and Durable Protective Immunity Against Heterologous Viruses

Seung Mo Jin, Ju Hee Cho, Yebin Seong, W.A. Gayan Chathuranga, Yejin Gwak, Young-Woock Noh, Min-Ho Lee, Sang-Seok Oh, Jin-Ho Choi, Jong-Soo Lee[**], *Yong Taik Lim*[*]

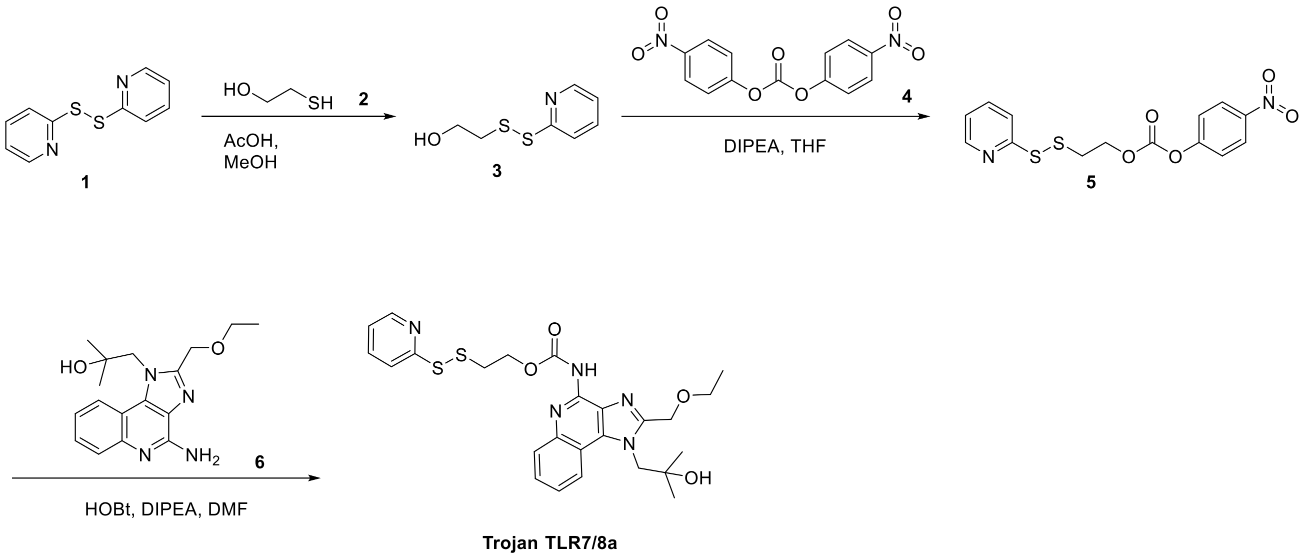

Figure S1. Schematic illustration of the synthesis of Transiently masked TLR7/8a (2-(pyridin-2-yl-disulfaneyl)ethyl (2-(ethoxymethyl)-1-(2-hydroxy-2-methylpropyl)-1H-imidazo[4,5-c]quinolin-4-yl)carbamate).

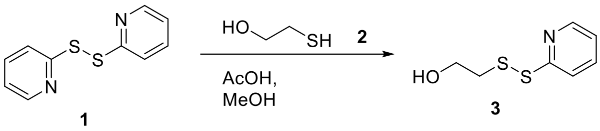

Figure S2. Synthesis and characterization of compound 3, 2-(pyridin-2-yldisulfanyl)ethanol. To a solution of 2-(pyridin-2-yldisulfanyl)pyridine (1) (35 g, 0.158 mol) in 250 ml of methanol, glacial acetic acid (6 ml) was added as a catalyst. A solution of 2-mercaptoethanol (2) (12 g, 0.153 mol, 11 ml) in 250 ml of methanol was then added dropwise to the above mixture at room temperature while stirring continuously. The reaction mixture was stirred at room temperature overnight. Subsequently, the solvent was evaporated, yielding the crude product as a yellow oil. The crude product was purified by flash column chromatography using silica gel as the stationary phase and a mixture of ethyl acetate and hexane as the eluent. The polarity of the eluent was increased using 40% ethyl acetate/hexane to isolate 2-(pyridin-2-yldisulfanyl)ethanol (25 g, 89%) as a light yellow oil. LC–MS: Rt =1.067 min, (ESI) m/z. [M+H]+ found, 188.0; $C_7H_9NOS_2$ theoretical, 187.01.

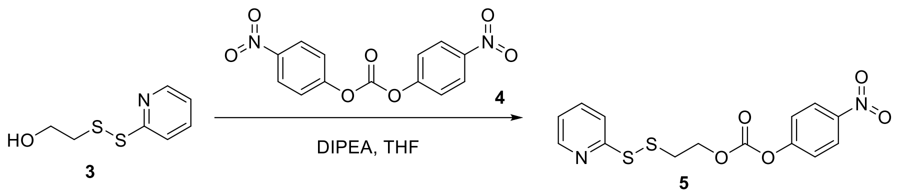

Figure S3. Synthesis and characterization of compound 5, (4-nitrophenyl) [2-(pyridin-2-yldisulfanyl)ethyl] carbonate. A solution of 2-(pyridin-2-yldisulfanyl)ethanol (3) (19 g, 0.1 mol), bis(4-nitrophenyl) carbonate (4) (46.32 g, 0.152 mol) and DIPEA (26 ml, 0.152 mol) in CH_2Cl_2 (500 ml) was placed under Ar protection. The mixture was stirred at room temperature for 5 h. The mixture was washed with water, and the organic phase was dried over $MgSO_4$. The organic solvent was evaporated under reduced pressure, and the residue was purified by flash chromatography (hexane/AcOEt 4:1 to 2:1) to obtain 4-nitrophenyl 2-(pyridin-2-yldisulfanyl)ethyl carbonate (11.21 g, 31.4%) as a light yellow oil. LC–MS: Rt =1.352 min, (ESI) m/z. [M+H]+ found, 353.7; $C_{14}H_{12}N_2O_5S_2$ theoretical, 352.02.

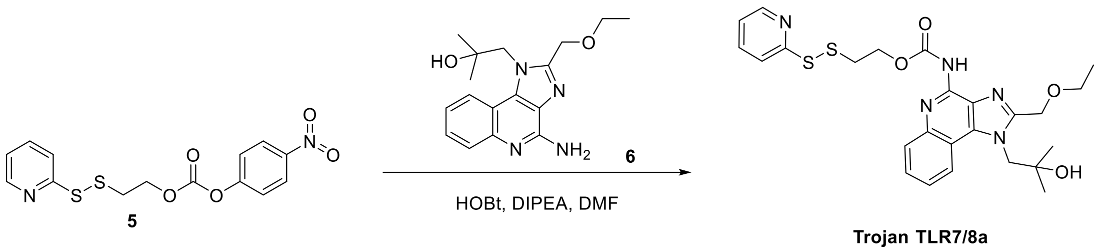

Figure S4. Synthesis and characterization of Transiently masked TLR7/8a, 2-(pyridin-2-yl-disulfaneyl)ethyl (2-(ethoxymethyl)-1-(2-hydroxy-2-methylpropyl)-1H-imidazo[4,5-c]quinolin-4-yl)carbamate. HOBt (3.22 g, 23.8 mmol), DIPEA (11 ml, 63.6 mmol), and 1-(4-amino-2-(ethoxymethyl)-1H-imidazo[4,5-c]quinolin-1-yl)-2-methylpropan-2-ol (6) (5.00 g, 15.9 mmol) were added to a solution of (4-nitrophenyl) [2-(pyridin-2-yldisulfanyl)ethyl] carbonate (5) (11.21 g, 31.8 mol) in DMF (100 ml). The mixture was stirred at 40 °C for 18 h. LC–MS showed that at this time, the starting material had been completely consumed, and one main peak with the desired mass was detected. The reaction mixture was concentrated under reduced pressure, and the residue was purified by silica gel chromatography by elution with MeOH in DCM (from 0 to 5%) over 10 min to give 2-(pyridin-2-yl-disulfaneyl)ethyl (2-(ethoxymethyl)-1-(2-hydroxy-2-methylpropyl)-1H-imidazo[4,5-c]quinolin-4-yl)carbamate (5.6 g, 67%) as a light yellow gum. LC–MS: Rt = 1.091 min, (ESI) m/z [M+H]+ found, 528.7; $C_{25}H_{29}N_5O_4S_2$ theoretical, 527.17. 1H NMR (400 MHz, CDCl3) δ 8.47 (d, J = 4.8 Hz, 1H), 8.22–8.13 (m, 2H), 7.75 (d, J = 8.0 Hz, 1H), 7.64 (dd, J = 19.2, 7.6 Hz, 2H), 7.51 (t, J = 7.6 Hz, 1H), 7.13–7.05 (m, 1H), 4.94 (s, 2H), 4.81 (s, 2H), 4.55 (t, J = 6.4 Hz, 2H), 3.67 (q, J = 7.2 Hz, 2H), 3.18 (t, J = 6.4 Hz, 2H), 1.35 (s, 6H), 1.25 (d, J = 6.8 Hz, 3H). The purity by HPLC was 98.37% (214 nm) and 98.25% (254 nm).

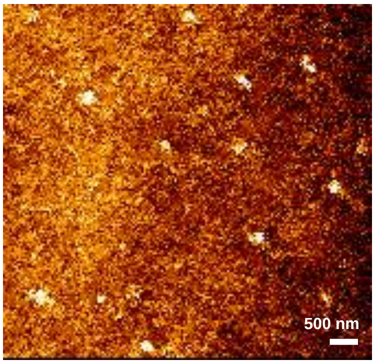

Figure S5. Atomic force microscopy (AFM) image of NP-TLR7/8a in dilution (1/400 X).

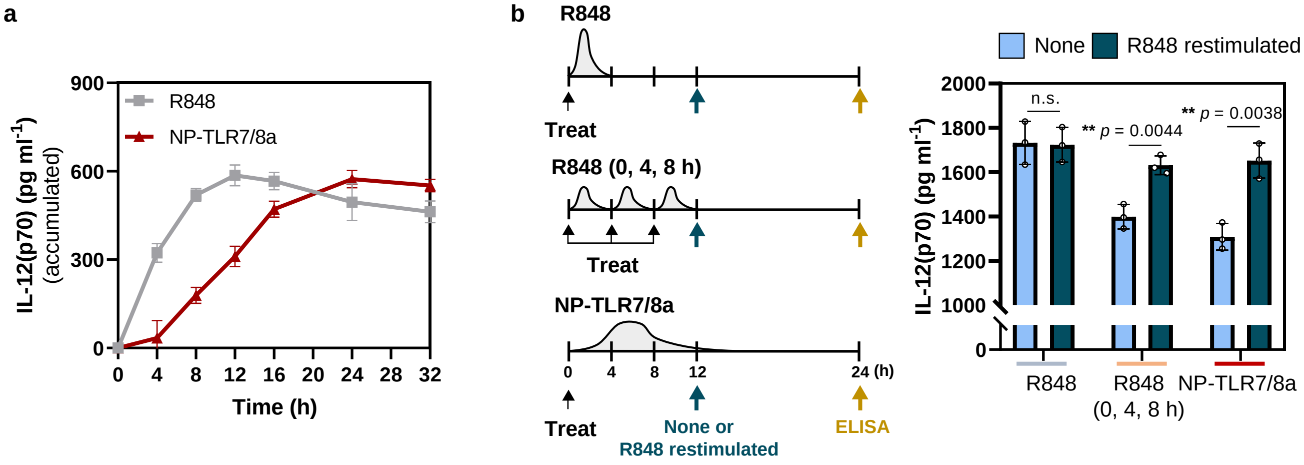

Figure S6. Dynamic cellular activation of NP-TLR7/8a and its suppression of TLR tolerance. a, Time-dependent cellular activation of R848 or NP-TLR7/8a. Indicated samples were treated on BMDCs and time-dependent IL-12(p70) levels were evaluated after their treatment (n = 3). **b,** TLR tolerance in BMDCs. Indicated samples were treated on BMDCs for 12 h and then restimulated or not with R848 (1 μg mL^{-1}). The amount of IL-12(p70) production was evaluated 24 h after the initial sample treatment (n = 3). All data are presented as the mean ± s.d. Statistical significance was evaluated by an unpaired two-tailed t-test in **b**. *P* values: NS, not significant; **P*<0.05, ***P*<0.01, ****P*<0.001, *****P*<0.0001.

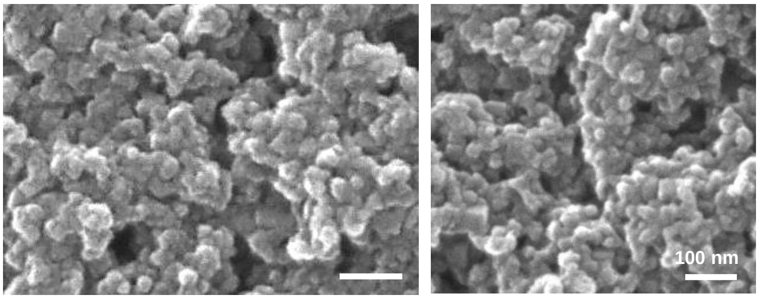

Figure S7. Scanning electron microscopy (SEM) images of the structure of IM-Gel (NP-TLR7/8a).

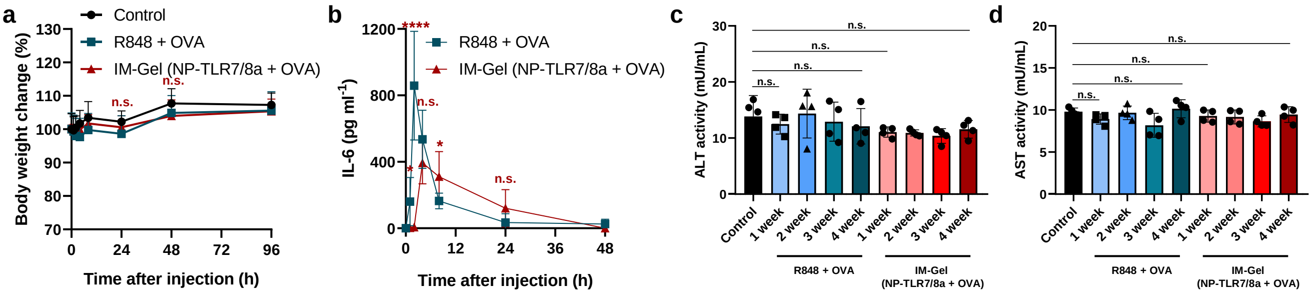

Figure S8. Short- and long-term biosafety analysis of IM-Gel after subcutaneous injection. a,b Short-term biosafety **(a)** body weight change (n = 6) and **(b)** serum IL-6 level (n = 6) analysis. **c,d** Long-term biosafety **(c)** ALT activity (n = 4) and **(d)** AST activity (n = 4) analysis after subcutaneous injection. All data are presented as the mean ± s.d. Statistical significance was evaluated by one-way ANOVA with Tukey's multiple comparison test in **a, c and d**. Statistical significance was evaluated by an unpaired two-tailed *t* test in **b**. *P* values: NS, not significant; *P<0.05, **P<0.01, ***P<0.001, ****P<0.0001.

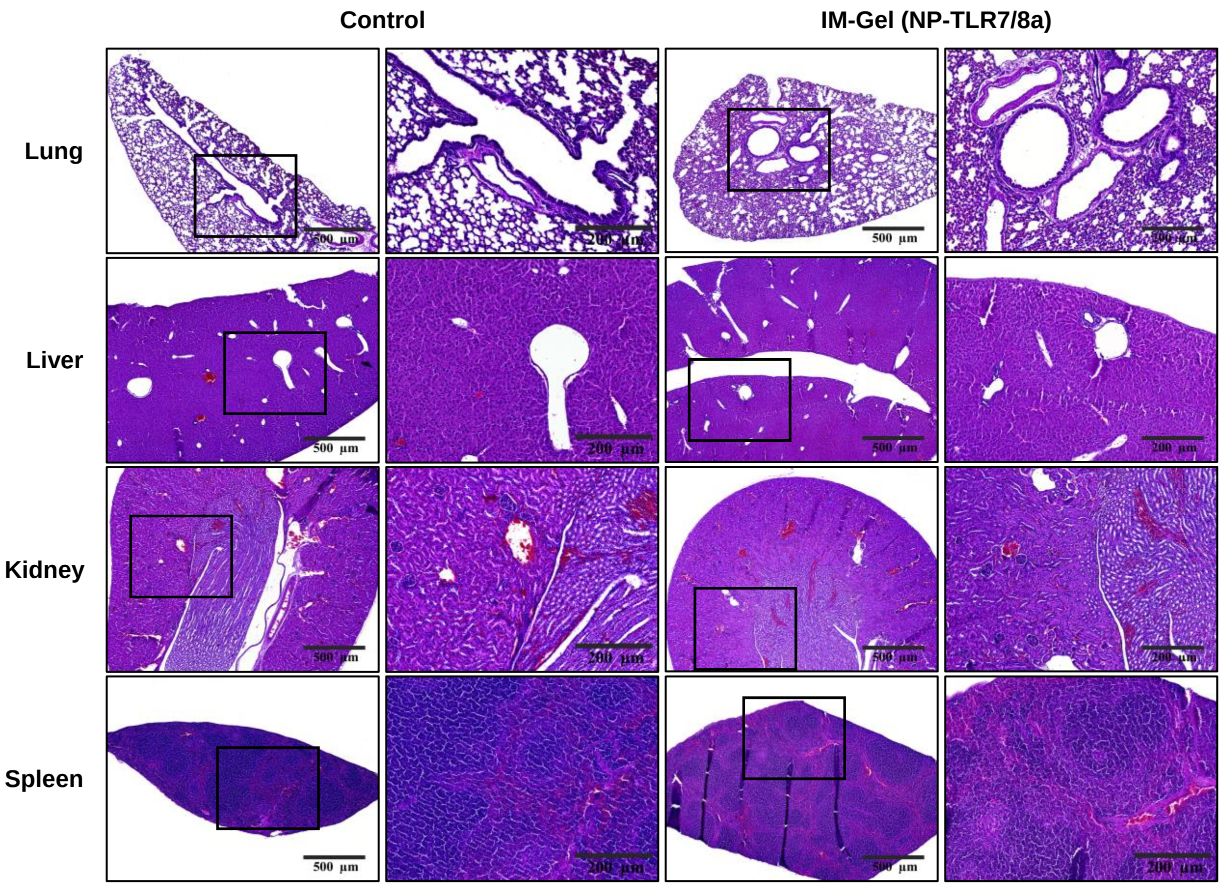

Figure S9. Histological examinations of different organs 7 days post-immunization of IM-Gel. Representative images of histological examinations of different organs (lung, liver, kidney, and spleen) 7 days post-immunization of IM-Gel.

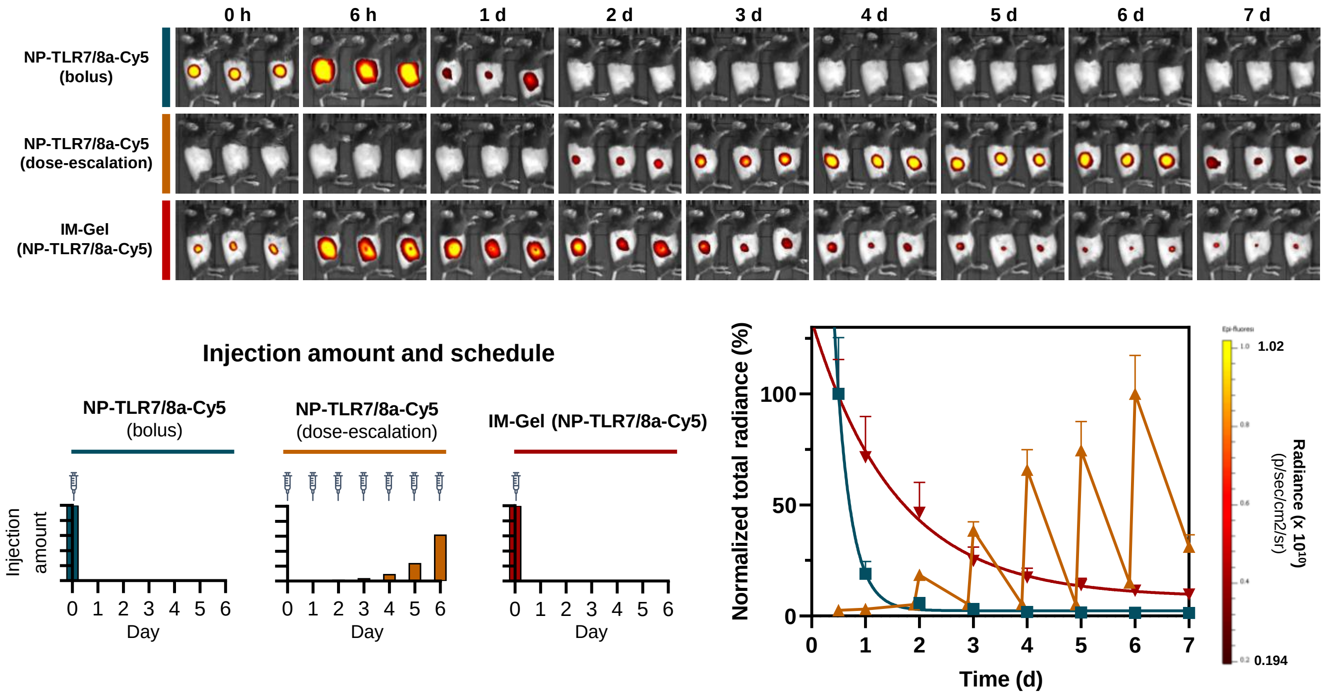

Figure S10. IVIS images and average radiance of fluorescent signal in the injection site over time after subcutaneous vaccination in wild-type C57BL/6 mice (n = 3).

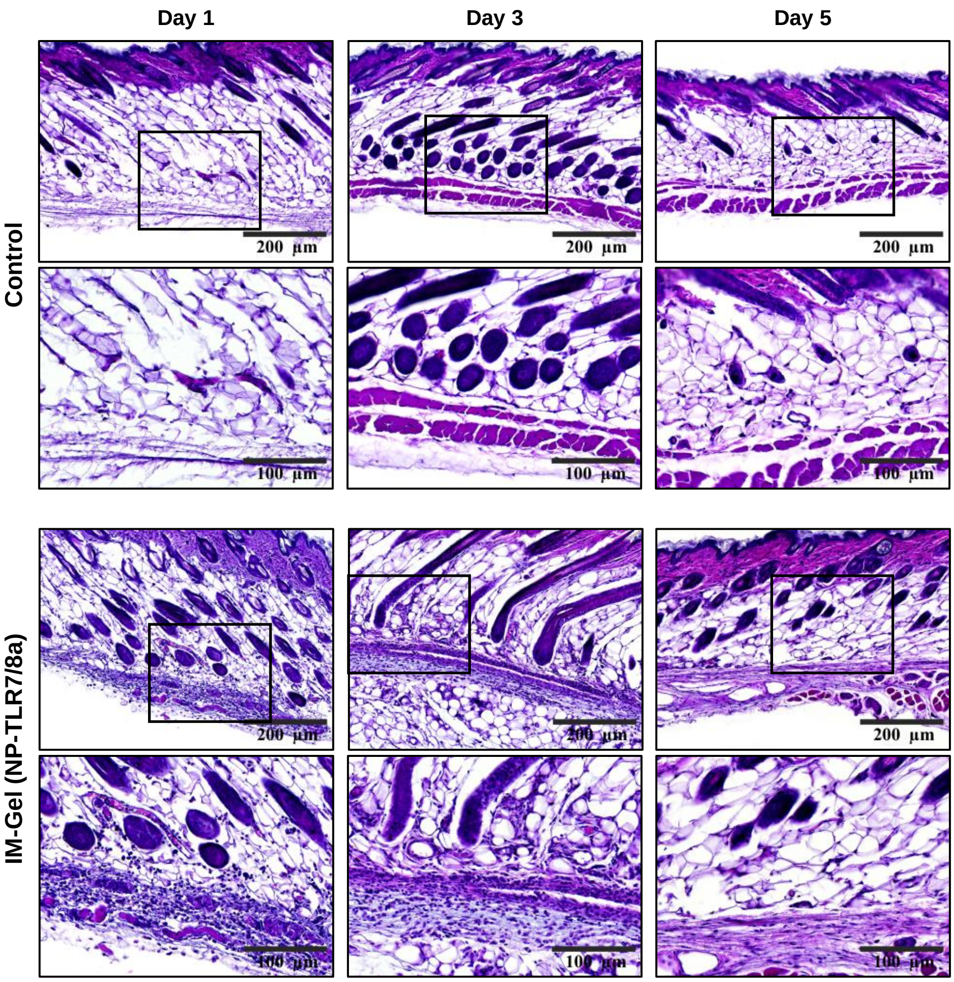

Figure S11. Histological examinations of skin after subcutaneous injection of IM-Gel (NP-TLR7/8a). Representative images of histological examinations of skin 1-, 3- or 5-days post-immunization of IM-Gel (NP-TLR7/8a).

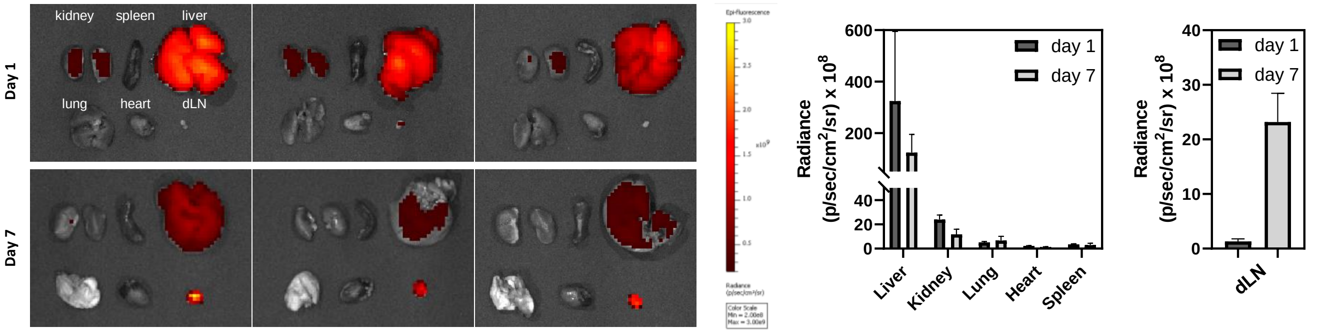

Figure S12. Time dependent biodistribution of IM-Gel in different organs after subcutaneous injection. Biodistribution of IM-Gel in different organs (liver, kidney, lung, heart, spleen, and draining lymph node) at 1 or 7 days after subcutaneous injection (n = 3).

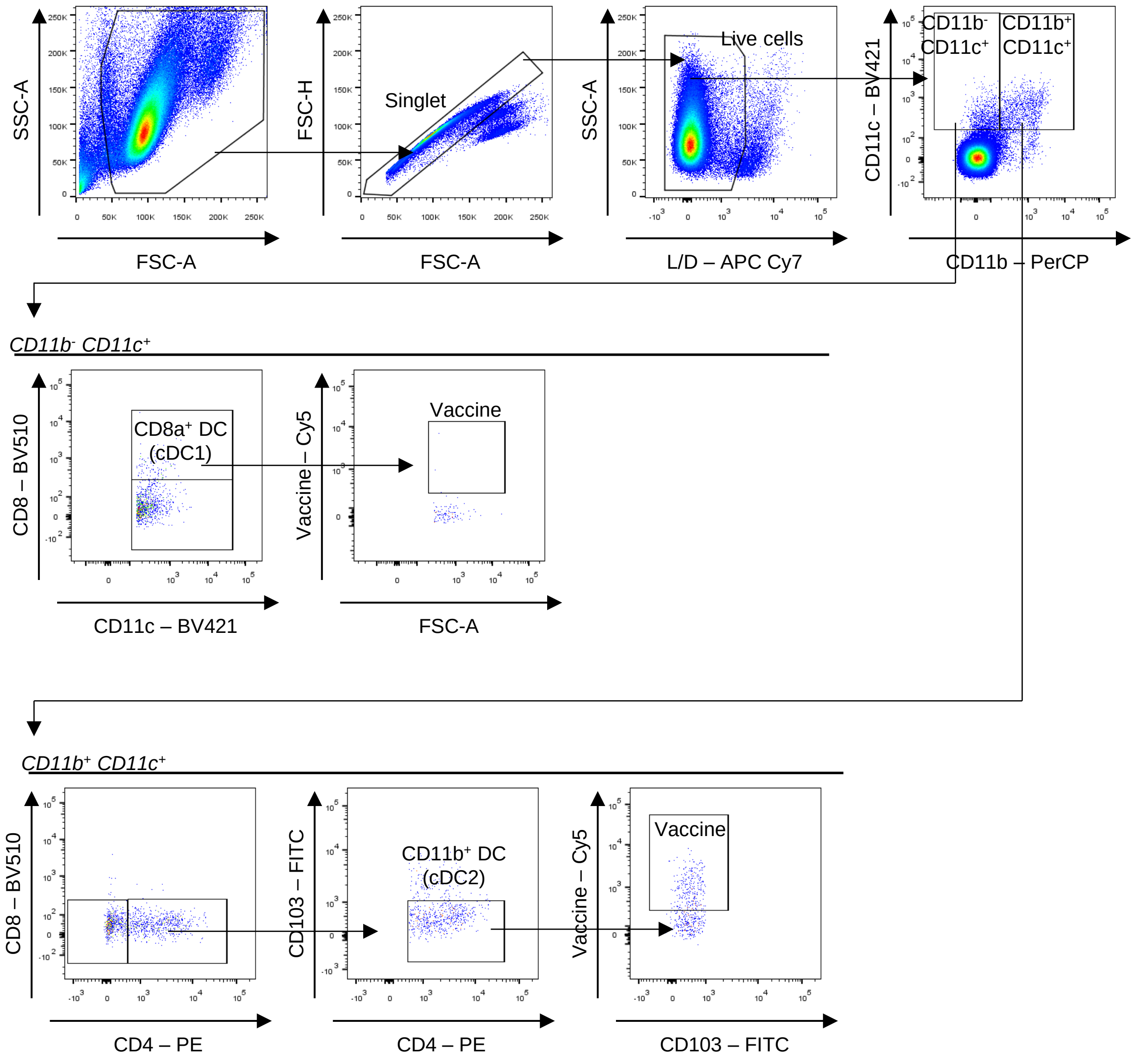

Figure S13. Flow cytometry gating strategy for Figure 2h, illustrating the kinetics of the percentage of vaccine^{+} APCs in the TDLN: cDC1s (CD11b^{-}CD11c^{+}CD8^{+}) and cDC2s (CD11b^{+}CD11c^{+}CD8^{-}CD4^{+}CD103^{-}). The gating strategy for all samples was set to remove large clumps or aggregates of cells (FSC-H and FSC-A gating), cell debris, and dead cells (live/dead gating).

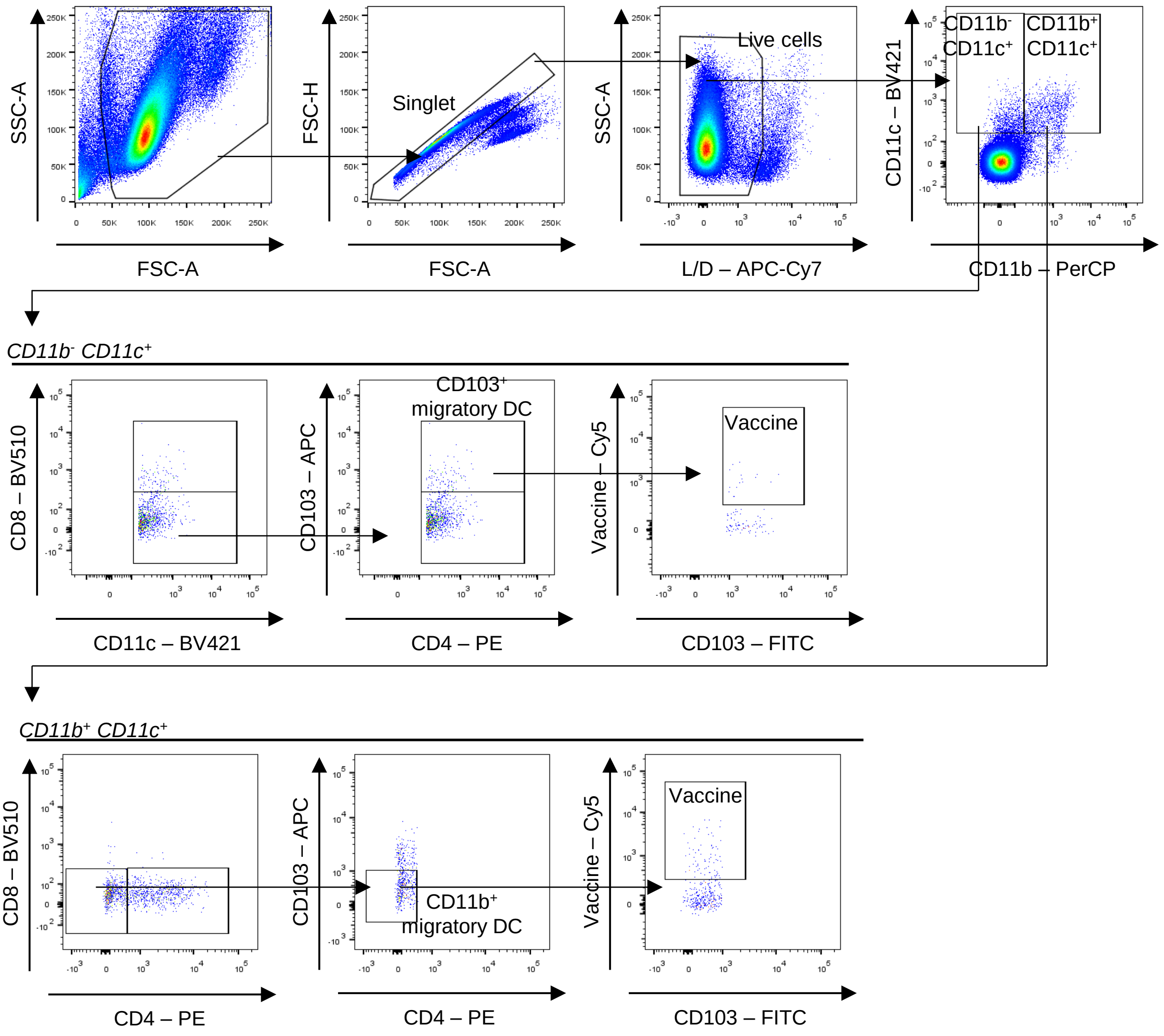

Figure S14. a) Flow cytometry gating strategy for Figure 2h, illustrating the kinetics of the percentage of vaccine^{+} APCs in the TDLN: CD103 migratory DCs (CD11b^{-}CD11c^{+}CD8^{-}CD4^{-}CD103^{+}) and CD11b migratory DCs (CD11b^{+} CD11c^{+} CD8^{-}CD4^{-}CD103^{-}). The gating strategy for all samples was set to remove large clumps or aggregates of cells (FSC-H and FSC-A gating), cell debris, and dead cells (live/dead gating).

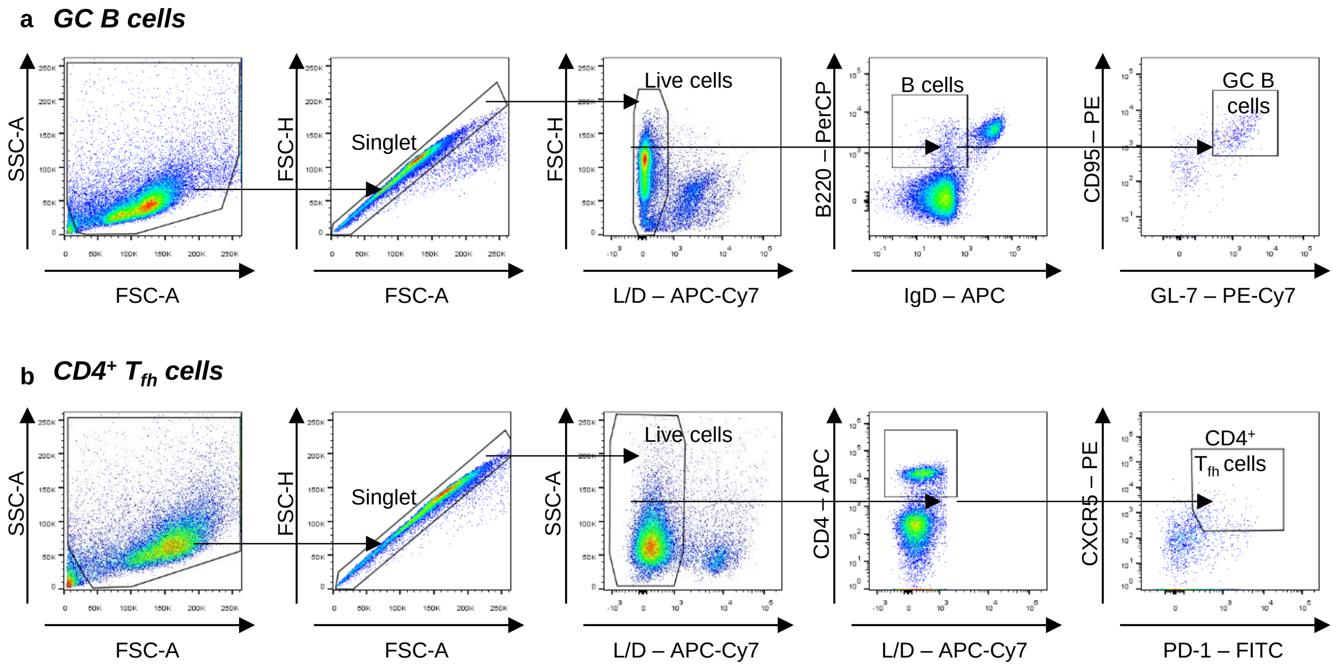

Figure S15. a-b) Flow cytometry gating strategy for Figure 4c, illustrating GC B cells **(a)** and follicular helper $CD4^+$ T cells **(b)**. The gating strategy for all samples was set to remove large clumps or aggregates of cells (FSC-H and FSC-A gating), cell debris, and dead cells (live/dead gating).

Ag-specific CD8$^+$ T cells

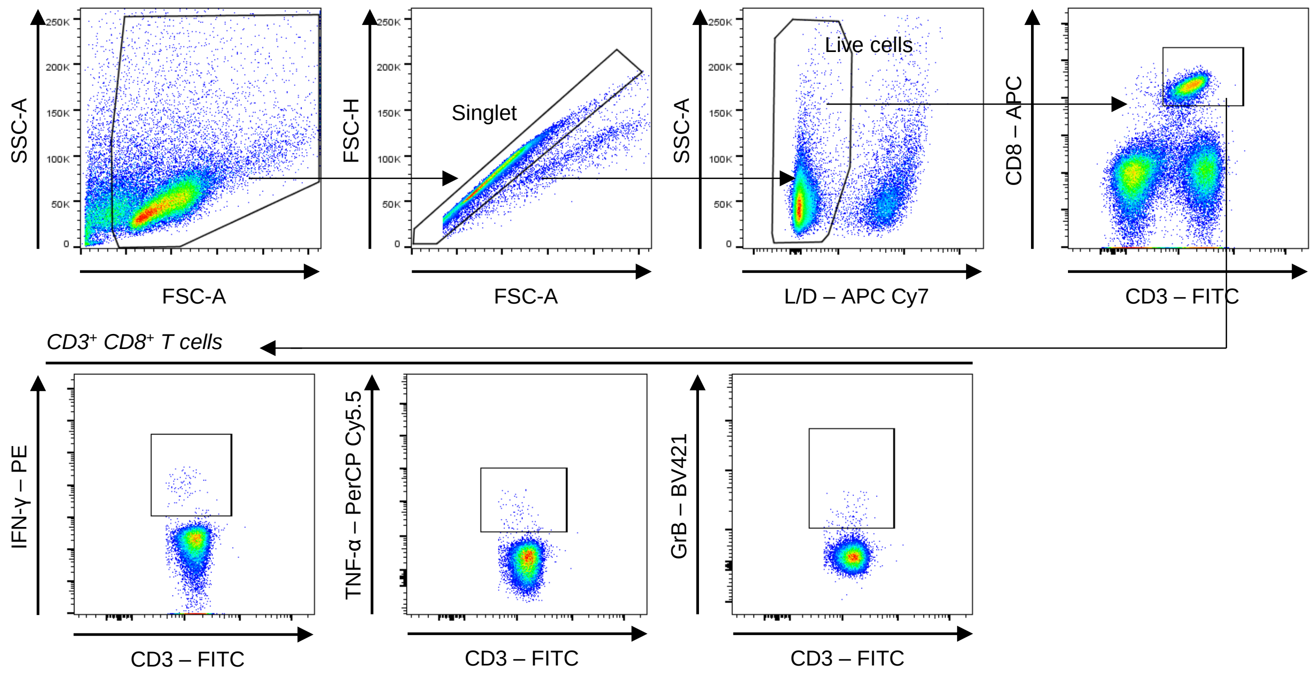

Figure S16. Flow cytometry gating strategy for Figure 4e, illustrating the analysis of immune cells *in vivo*. Populations of IFN-γ$^+$-, TNF-α$^+$- or GrB$^+$-producing CD8$^+$ T cells (CD3$^+$CD8$^+$) in the draining lymph node. The gating strategy for all samples was set to remove large clumps or aggregates of cells (FSC-H and FSC-A gating), cell debris, and dead cells (live/dead gating).

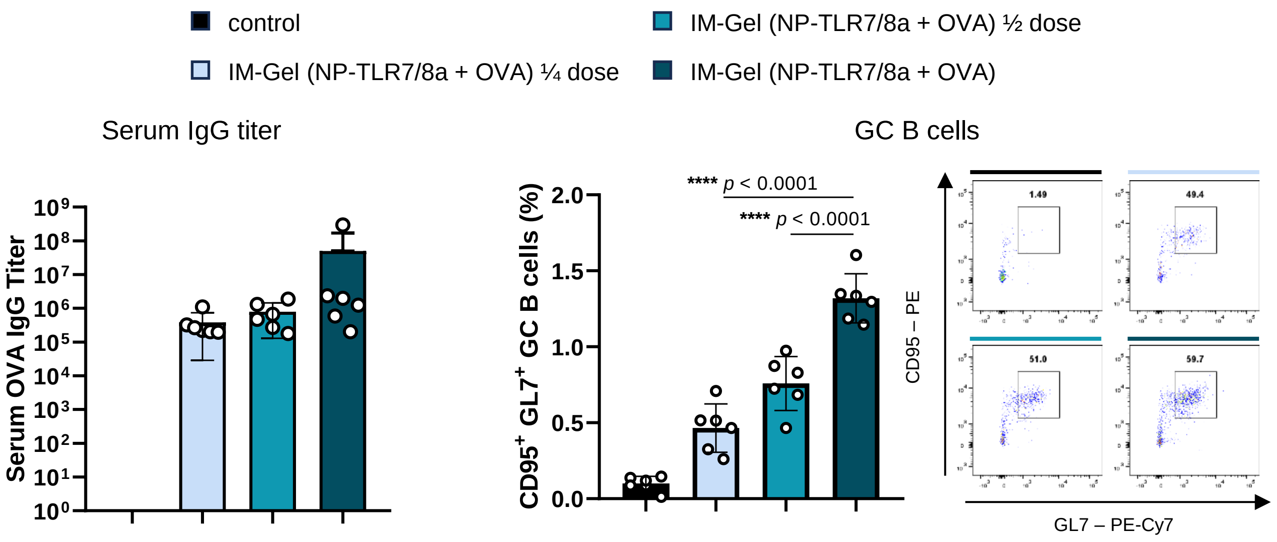

Figure S17. Dose-dependency of IM-Gel. Serum OVA IgG titers (left) and the population of GC B cells in the dLN (right) were analyzed two weeks after two immunizations at a two-week interval (n = 6). All data are presented as the mean ± s.d. Statistical significance was evaluated by one-way ANOVA with Tukey's multiple comparison test. *P* values: NS, not significant; **P*<0.05, ***P*<0.01, ****P*<0.001, *****P*<0.0001.

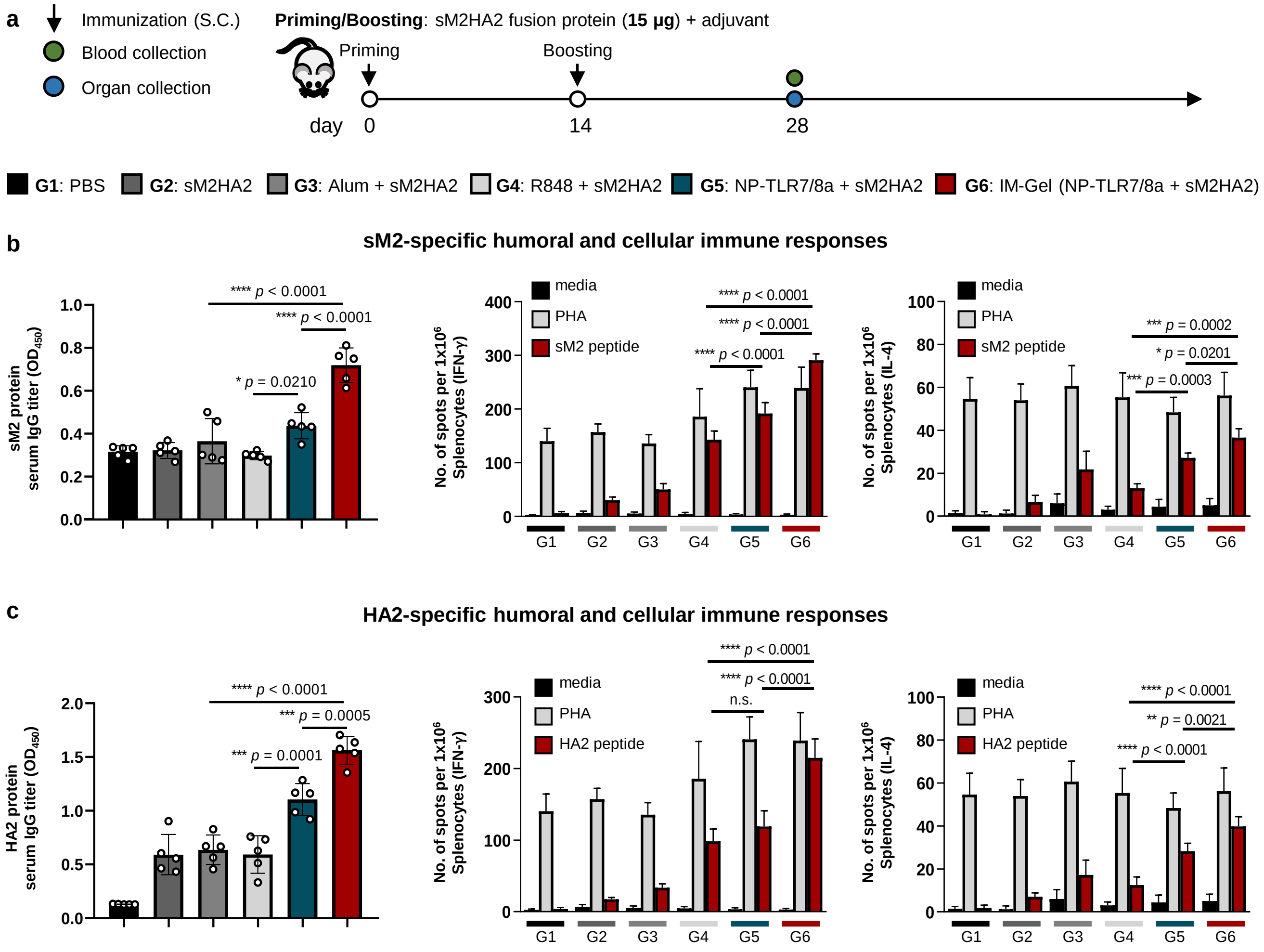

Figure S18. sM2- or HA2-specific humoral and cellular immune responses after immunization with sM2HA2 protein and adjuvant. a) Injection and analysis schedule and dose of sM2HA2 fusion protein vaccination with adjuvant. Indicated samples (sM2HA2 protein 15 µg, TLR7/8a 30 nmol) were injected two times with a 14-day interval. **b)** sM2-specific IgG, IgG1, and IgG2a titer (n = 5) and number of spots of IFN-γ- or IL-4-secreting splenocytes after sM2 peptide restimulation determined uing the ELISPOT assay (n = 5). **c)** HA2-specific IgG, IgG1, and IgG2a titer (n = 5) and number of spots of IFN-γ- or IL-4-secreting splenocytes after HA2 peptide restimulation determined uing the ELISPOT assay (n = 5). All data are presented as the mean ± s.d. Statistical significance was evaluated by one-way ANOVA with Tukey's multiple comparison test in **b and c**. *P* values: NS, not significant; **P*<0.05, ***P*<0.01, ****P*<0.001, *****P*<0.0001.

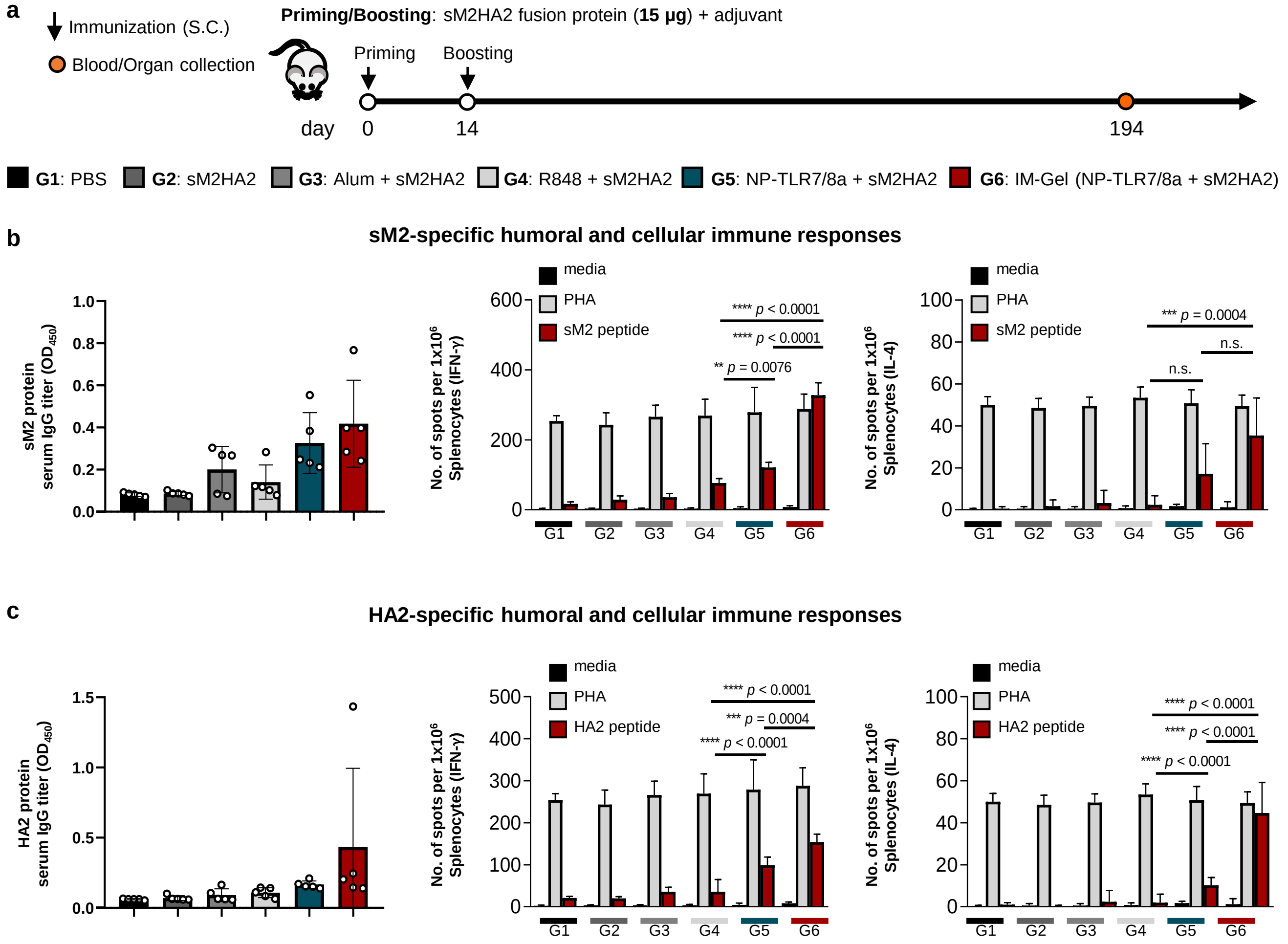

Figure S19. sM2- or HA2-specific long lasting humoral and cellular immune responses 6 months after immunization with sM2HA2 protein and adjuvant. a) Injection and analysis schedule and dose of sM2HA2 fusion protein vaccination with adjuvant. Indicated samples (sM2HA2 protein 15 μg, TLR7/8a 30 nmol) were injected two times with a 14-day interval. **b)** sM2-specific IgG, IgG1, and IgG2a titer (n = 5) and number of spots of IFN-γ- or IL-4-secreting splenocytes after sM2 peptide restimulation determined uing the ELISPOT assay (n = 5). **c)** HA2-specific IgG, IgG1, and IgG2a titer (n = 5) and number of spots of IFN-γ- or IL-4-secreting splenocytes after HA2 peptide restimulation determined uing the ELISPOT assay (n = 5). All data are presented as the mean ± s.d. Statistical significance was evaluated by one-way ANOVA with Tukey's multiple comparison test in **b and c**. *P* values: NS, not significant; **P*<0.05, ***P*<0.01, ****P*<0.001, *****P*<0.0001.

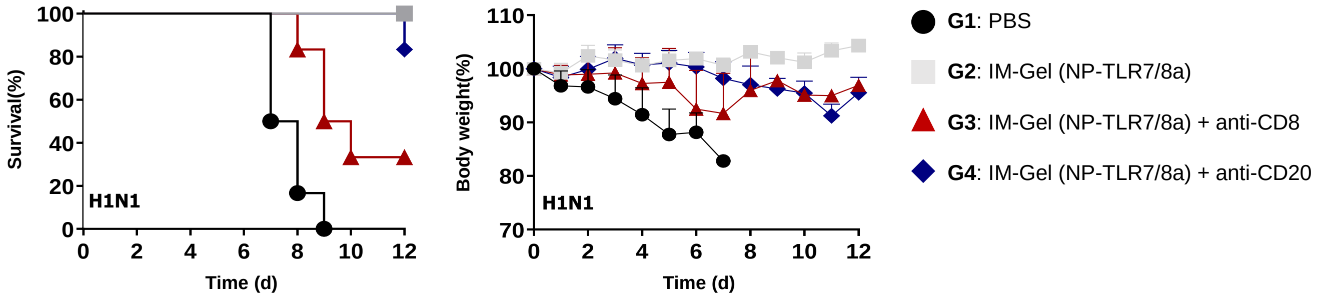

Figure S20. Leading role of cellular or humoral immunity after IM-Gel vaccination. Challenge experiments in the condition of antibody mediated immune cell depletion. Survival rate (left) and the body weight change (right) were analyzed during 12 days after infection with H1N1 after two immunizations at a two-week interval (n = 6).

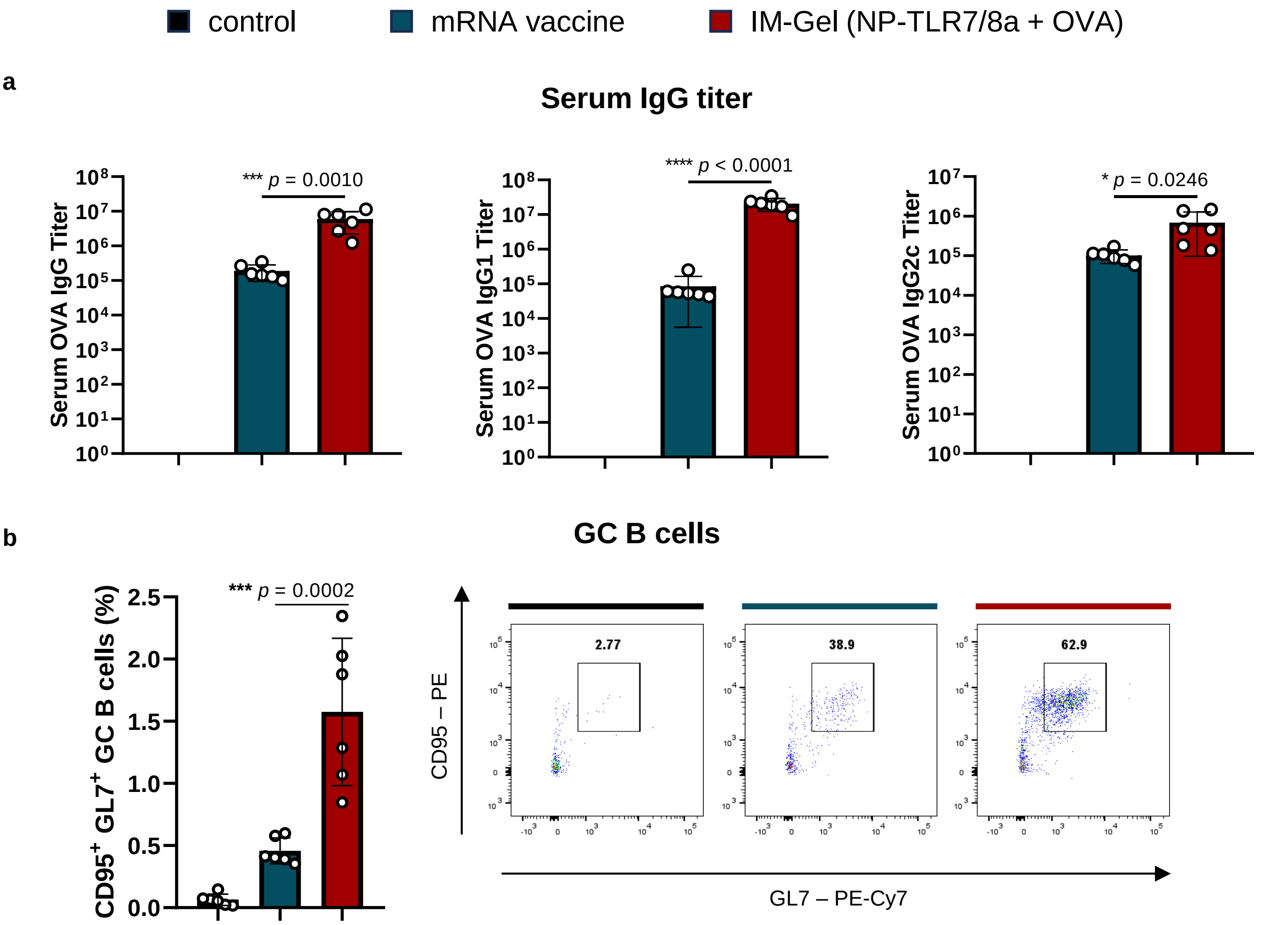

Figure S21. Vaccination efficacy comparison of IM-Gel with conventional mRNA vaccine platform. mRNA vaccine (OVA mRNA 5 μg) and IM-Gel (NP-TLR7/8a + OVA) (OVA 10 μg, TLR7/8a 30 nmol) were subcutaneously administered two times at a two-week interval (n = 6). **a)** Serum OVA-specific total IgG, IgG1, and IgG2c titers and the **b)** population of GC B cells in the dLN were analyzed two weeks after second immunization (n = 6). All data are presented as the mean ± s.d. Statistical significance was evaluated by one-way ANOVA with Tukey's multiple comparison test. *P* values: NS, not significant; **P*<0.05, ***P*<0.01, ****P*<0.001, *****P*<0.0001.

Antibody	Manufacturer	Clone	Catalog number
Flow Cytometry			
FITC anti-mouse CD3	BioLegend	17A2	100204
Alexa Flour 488 anti-mouse CD3	BioLegend	17A2	100212
APC anti-mouse CD4	BioLegend	GK1.5	100412
PE anti-mouse CD4	BioLegend	RM4-5	100512
APC anti-mouse CD8a	BioLegend	53-6.7	100712
Brilliant Violet 510™ anti-mouse CD8a	BioLegend	53-6.7	100752
PE anti-mouse IFN-γ	BioLegend	W18272D	163504
Brilliant Violet 421™ anti-human/mouse Granzyme B	BioLegend	QA18A28	396414
PerCP/Cyanine5.5 anti-mouse TNF-α	BioLegend	MP6-XT22	506322
FITC anti-mouse CD279 (PD-1)	BioLegend	29F.1A12	135214
PerCP anti-mouse/human CD11b	BioLegend	M1/70	101230
Brilliant Violet 421™ anti-mouse CD11c	BioLegend	N418	117330
FITC anti-mouse CD103	BioLegend	2E7	121419
PerCP anti-mouse/human CD45R/B220	BioLegend	RA3-6B2	103234
Alexa Flour 594 anti-mouse/human CD45R/B220	BioLegend	RA3-6B2	103254
APC anti-mouse IgD	BioLegend	11-26c.2a	405714
PE anti-mouse CD95 (Fas)	BioLegend	SA367H8	152608
PE/Cyanine7 anti-mouse/human GL7 Antigen	BioLegend	GL7	144620
Biotin anti-mouse CD185 (CXCR5)	BioLegend	L138D7	145510
Depletion antibody			
Anti-mouse CD20	BioXCell	MB20-11	BE0356
Anti-mouse CD8a	BioXCell	2.43	BE0061

Table S1. List of antibodies used for flow cytometry analysis.

Protein	Amino acid position	Amino acid sequence
HA2	19-48	GYAADLKSTQNAIDEITNKVNSVIEKMNTQ
M2	2-16	SLLTEVETPTRNEWE

Table S2. List of peptide sequence for ELISPOT. Protein indicates the A/EM/Korea/W149/06 (H5N1) virus.
